# Second-Generation *Drosophila* Chemical Tags: Sensitivity, Versatility, and Speed

**DOI:** 10.1534/genetics.116.199281

**Published:** 2017-02-14

**Authors:** Ben Sutcliffe, Julian Ng, Thomas O. Auer, Mathias Pasche, Richard Benton, Gregory S. X. E. Jefferis, Sebastian Cachero

**Affiliations:** * Division of Neurobiology, MRC Laboratory of Molecular Biology, Cambridge CB2 0QH, UK; † Department of Zoology, University of Cambridge, CB2 3EH, UK; ‡ Center for Integrative Genomics, Faculty of Biology and Medicine, University of Lausanne, CH-1015, Switzerland; § Division of Cell Biology, MRC Laboratory of Molecular Biology, Cambridge CB2 0QH, UK

**Keywords:** immunohistochemistry, chemical labeling, chemical tags, neural circuits, protein labeling, fluorescence microscopy

## Abstract

Labeling and visualizing cells and subcellular structures within thick tissues, whole organs, and even intact animals is key to studying biological processes. This is particularly true for studies of neural circuits where neurons form submicron synapses but have arbors that may span millimeters in length. Traditionally, labeling is achieved by immunofluorescence; however, diffusion of antibody molecules (>100 kDa) is slow and often results in uneven labeling with very poor penetration into the center of thick specimens; these limitations can be partially addressed by extending staining protocols to over a week (*Drosophila* brain) and months (mice). Recently, we developed an alternative approach using genetically encoded chemical tags CLIP, SNAP, Halo, and TMP for tissue labeling; this resulted in >100-fold increase in labeling speed in both mice and *Drosophila*, at the expense of a considerable drop in absolute sensitivity when compared to optimized immunofluorescence staining. We now present a second generation of UAS- and LexA-responsive CLIPf, SNAPf, and Halo chemical labeling reagents for flies. These multimerized tags, with translational enhancers, display up to 64-fold increase in sensitivity over first-generation reagents. In addition, we developed a suite of conditional reporters (4xSNAPf tag and CLIPf-SNAPf-Halo2) that are activated by the DNA recombinase Bxb1. Our new reporters can be used with weak and strong GAL4 and LexA drivers and enable stochastic, intersectional, and multicolor Brainbow labeling. These improvements in sensitivity and experimental versatility, while still retaining the substantial speed advantage that is a signature of chemical labeling, should significantly increase the scope of this technology.

## Abstract (precis)

Thick tissue specimens present major challenges for labeling cells and subcellular structures in a rapid and reliable manner. Sutcliffe *et al.* present...

VISUALIZING molecules in intact tissues with high sensitivity and specificity is of paramount importance in many fields of biological research. Traditionally, cellular and subcellular labeling has depended on immunostaining that combines primary antibodies specific to a molecule of interest, followed by labeled secondary antibodies. Recently we and others have adapted chemical labeling approaches that were initially developed for *in vitro* or single-cell studies (Keppler *et al.* 2003; Gautier *et al.* 2008; Los *et al.* 2008) for use in genetically defined cells within intact fly and mouse tissues (Kohl *et al.* 2014; Yang *et al.* 2015). These overcame a fundamental limitation of antibodies: low diffusion rate that causes poor penetration of thick tissue samples. The basic principle of chemical labeling is the use of small protein tags (engineered from enzymes) that can covalently and irreversibly bind small molecule substrates [for a schematic and structure of the substrates see Figure 1A in Kohl *et al.* (2014)]. These substrates can be conjugated with a variety of labels such as fluorophores for light microscopy and colloidal gold for electron microscopy (Keppler *et al.* 2003; Gautier *et al.* 2008; Vistain *et al.* 2016). High-efficiency binding in combination with small substrate size allows easy tissue penetration and fast quantitative staining (Kohl *et al.* 2014).

**Figure 1:**
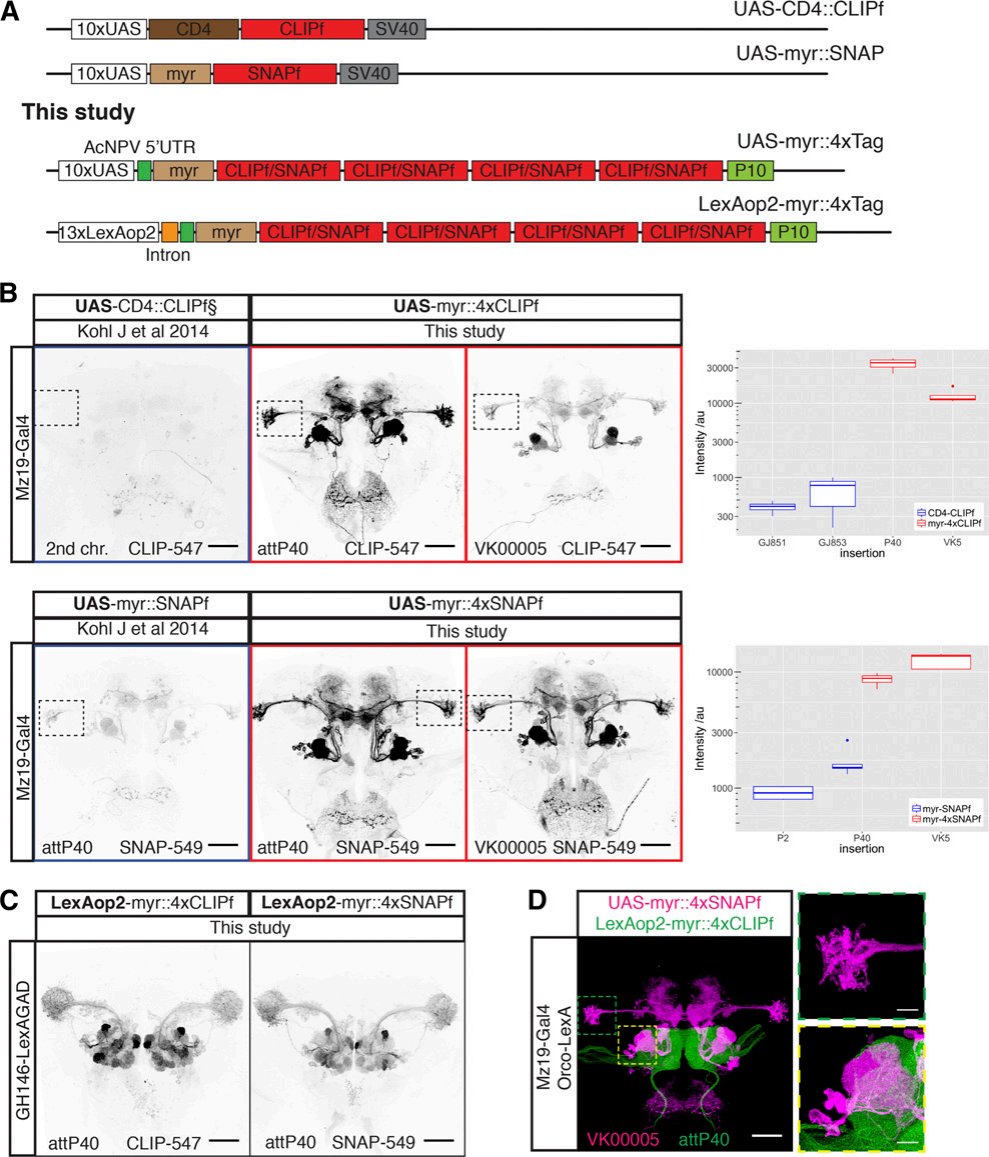
New CLIPf and SNAPf reporters have increased sensitivity. (A) Schematic of previous CLIPf/SNAPf reporters from Kohl *et al.* (2014) and the new reporters from this study. (B) Labeling of Mz19-Gal4 neurons using the old and new reporters. Each panel contains information on the dye used and insertion sites. Dotted boxes in images highlight the Lateral Horn region used for quantification. Boxplots show the quantification of fluorescence intensity of the axonal terminals of projection neurons in the lateral horn (arbitrary units). Boxplot *n* numbers were: GJ853 CD4::CLIPf on the second chromosome *n* = 3, GJ851 CD4::CLIPf on the third chromosome *n* = 4, P40 myr::4xCLIPf *n* = 4, VK00005 myr::4xCLIPf *n* = 4, P40 myr::4xSNAPf *n* = 4, VK00005 myr::4xSNAPf *n* = 5, P2 myr::SNAPf *n* = 4, and P40 myr::SNAPf *n* = 5. (C) New LexAop2-myr::4xCLIPf/4xSNAPf reporters labeling olfactory projection neuron using the weak GH146-LexA::GAD driver. (D) Orthogonal labeling of olfactory sensory neurons (green) and projection neurons (magenta) using new tags. Shown is the max intensity projection of a confocal stack after deconvolution. Images in B and C were acquired using the same microscope settings. Bars, 50 μm in whole brain images and 10 μm in higher magnification images of the boxed areas in D.

Improvements in speed and penetration achieved by the first generation of chemical labeling reagents are particularly important in neural circuit research where labeling of neurons in deep structures within intact brains is essential for understanding connected networks in the brain but experimentally very challenging. To illustrate this point, optimal immunostaining of a fly brain takes more than a week (Ostrovsky *et al.* 2013) while a mouse brain can take months even when combined with tissue clearing methods (Chung *et al.* 2013). By contrast, multicolor chemical labeling of a fly brain can be completed within 1 hr, with <10 min of staining time. Other important advantages of chemical labeling reagents are that they reduce off-target labeling and as completely synthetic reagents, in contrast to antibodies, they are not produced using animals. In comparison to the use of genetically encoded fluorescent proteins, simply by changing the substrate, reporter lines with chemical labeling transgenes enable rapid testing and switching to new fluorophores with properties required for constantly evolving imaging modalities.

While the published *Drosophila* reagents offer unparalleled staining speed (Kohl *et al.* 2014), they produce considerably weaker signal than traditional immunolabeling of genetically encoded reporters, limiting their use to relatively strong Gal4 driver lines (Brand and Perrimon 1993). We now introduce a second generation of fly reagents with greatly increased sensitivity. Furthermore, we have increased the versatility of the system by developing reporters for the LexA-based expression system (Lai and Lee 2006) and reagents for conditional and stochastic labeling based on Bxb1 DNA recombinase (Huang *et al.* 2011). Finally, we show the utility of chemical labeling in targeting challenging tissues such as the fly antennae. We expect these new tools will greatly increase the use of chemical labeling within the research community, especially speeding up projects that require large numbers of stainings.

## Materials and Methods

### Drosophila stocks

Fly stocks were maintained at 25° on iberian food. The driver lines used in this study are MZ19-Gal4 (Ito *et al.* 1998), MB247-Gal4 (FlyBaseID: FBst0050742), Fru-Gal4 (Stockinger *et al.* 2005), BG57-Gal4 (FlyBaseID: FBst0032556), GMR50A02-Gal4 (FlyBaseID: FBti0136386), GMR54F05-Gal4 (FlyBaseID: FBst0039080), GMR59F02-Gal4 (FlyBaseID: FBst0039221), OR22a-Gal4 (Vosshall *et al.* 2000), IR84a-Gal4 (Silbering *et al.* 2011), Orco-LexA::VP16 (Lai and Lee 2006), GH146-LexA::GAD (Lai *et al.* 2008), nSyb-LexA::P65 in attP40 (Pfeiffer *et al.* 2012), and MB247-LexA (Pitman *et al.* 2011). The reporter lines used in this study are UAS-CD4::CLIPf on second and third chromosomes, UAS-myr::SNAPf in attP40 and attP2, UAS-myr::Halo2 in attP40 (Kohl *et al.* 2014); for details of the new reporter lines generated in this study see Supplemental Material, Table S1 in File S1. All images are of female brains, apart from the brains in Figure 4D which are male; all flies were dissected 3–4 days after eclosion.

### Drosophila constructs and transgenic flies

*Drosophila* transformation plasmids from Table 1 were made by Gibson assembly (Gibson *et al.* 2009) (Figures S4 to S13 in File S1) or restriction enzyme cloning (Figures S14 to S20 in File S1) and deposited at Addgene. Figures S4 to S20 in File S1 show the primers and enzymes used to make each plasmid. Transgenic flies were made by BestGene and deposited at Bloomington (see http://flystocks.bio.indiana.edu/Browse/misc-browse/chemtag.php). The background expression for the landing sites used in this study (*i.e.*, expression in the absence of LexA or Gal4) has been shown to be minimal (Pfeiffer *et al.* 2010).

**Table 1:** *Drosophila* transformation plasmids

Plasmid name	GeneBank accession no.	Addgene ID	Cloning schematic
UAS-myr::4xCLIPf	KY511544	87635	Figure S6 in File S1
LexAop2-myr::4xCLIPf	KY511545	87636	Figure S8 in File S1
UAS-myr::4xSNAPf	KY511546	87637	Figure S7 in File S1
LexAop2-myr::4xSNAPf	KY511547	87638	Figure S9 in File S1
UAS-myr::>HA-Bxb1.STOP > myr::4xSNAPf	KY511548	87639	Figure S10 in File S1
LexAop2-myr::>HA-Bxb1.STOP > myr::4xSNAPf	KY511549	87640	Figure S11 in File S1
HeatShock-Bxb1-SV40	KY511550	87641	Figure S4 in File S1
HeatShock-Bxb1	KY511551	87642	Figure S12 in File S1
UAS->FlpSTOP > Bxb1	KY511552	87643	Figure S13 in File S1
UAS > Bxb1	KY511553	—	Figure S13 in File S1
UAS->Bxb1.STOP > SNAPf > CLIPf > Halo2	KY511554	87644	Figure S5 in File S1
UAS-Halo7::CAAX	KY511555	87645	Figure S14 in File S1
UAS-3xHalo7::CAAX	KY511556	87646	Figure S15 in File S1
UAS-7xHalo7::CAAX	KY511557	87647	Figure S16 in File S1
UAS-Syt::Halo7	KY511558	87648	Figure S17 in File S1
UAS-3xSyt::Halo7	KY511559	87649	Figure S18 in File S1
UAS-7xSyt::Halo7	KY511560	87650	Figure S19 in File S1
UAS-LA::Halo2	KY511561	87651	Figure S20 in File S1

### Labeling reagents

Substrates were acquired either as stock solutions (*e.g.*, HaloTag TMR) or in powdered form (SNAPf and CLIPf substrates) and diluted/dissolved in anhydrous dimethyl sulfoxide (DMSO) (Life Technologies) to a concentration of 1 mM. Aliquots (5 μl) were stored at −20° in the presence of desiccant. We observed that using old DMSO or storing dissolved substrates in moist and/or warm conditions can lead to hydrolysis, reducing labeling efficiency. For a list of all substrates used in this study, see Table 2.

**Table 2:** Chemical tagging substrates used in this study

Substrate (abbreviation)	Fluorophore	Ex	Em	Binds to	Cell permeable	Supplier	Cat. #
SNAP-Cell 647-SiR (SNAP-SiR)	SiR	645	661	SNAPm/f	Yes	New England Biolabs	S9102S
SNAP-Surface 549 (SNAP-549)	Dyomics DY-549P1	560	575	SNAPm/f	No	New England Biolabs	S9112S
CLIP-Surface 488 (CLIP-488)	ATTO-TEC 488	506	526	CLIPm/f	No	New England Biolabs	S9232S
CLIP-Surface 547 (CLIP-547)	Dyomics DY-547	554	568	CLIPm/f	No	New England Biolabs	S9233S
HaloTag TMR Ligand (Halo-TMR)	TMR	555	585	Halo2/7	Yes	Promega	G8252
HaloTag SiR Ligand (Halo-SiR)	SiR	645	661	Halo2/7	Yes	K. Johnsson	n/a

Commercially available, fluorophore-coupled substrates for SNAP-, CLIP-, and Halo- are listed.

### Protocol for labeling Drosophila brains

Single and double channel labeling of *Drosophila* brains was carried out as previously described (Kohl *et al.* 2014). For labeling of UAS-LA::Halo2 fillet preparation of wandering third instar larvae were made followed by the same protocol used for labeling whole brains. For detailed information on staining Chemical Brainbow brains and antennal segments, see Supplemental *Materials and Methods* in File S1. We find that CLIPf substrates weakly bind SNAPf tag; therefore, if labeling both SNAPf and CLIPf in the same specimen, we recommend doing sequential SNAPf substrate incubation (minimum 5 min) then addition of CLIPf substrate (minimum 5 min) to avoid cross-reactivity.

### Image acquisition and deconvolution

Confocal stacks of fly brains were imaged at 768 × 768 pixels every 1 μm (voxel size of 0.46 × 0.46 × 1 μm; 0.6 zoom factor) using an EC Plan-Neofluar 40×/1.30 Oil DIC M27 objective and 16-bit color depth. Higher magnification images of cell bodies were acquired at 2048 × 2048 pixels every 0.45 μm (voxel size 0.1 × 0.1 × 0.45 μm; 1.0 zoom factor) using a Plan-Apochromat 63×/1.40 Oil DIC M27 objective and 16-bit color depths. Antennae were imaged at 1024 × 1024 pixels every 1 μm (voxel size 0.20 × 0.20 × 1 μm; 1.0 zoom factor) using an EC Plan-Neofluar 40×/1.30 Oil DIC M27 objective and 8-bit color depths. The image of the entire larval musculature (Figure 5B) was acquired as a tile scan with total dimensions 1536 × 2304 pixels every 1.0 μm (voxel size 1.84 × 1.84 × 1.0 μm; 0.6 zoom factor) with EC Plan-Neofluar 10×/0.30 M27 objective and 16-bit color depths. The high-magnification larval muscle inset was acquired at 2156 × 2156 pixels every 0.45 μm (voxel size 0.1 × 0.1 × 0.45 μm; 1.0 zoom factor) using a Plan-Apochromat 63×/1.40 Oil DIC M27 objective and 16-bit color depth. All images were acquired on a Zeiss LSM710 confocal microscope.

The confocal stack of the fly brain in Figure 1D was acquired using a Leica SP8 confocal microscope, following the Nyquist criterion, at 4224 × 4224 pixels every 0.313 μm (voxel size 0.076 × 0.076 × 0.313 μm; 0.9 zoom factor) using a HC PL APO CS2 40×/1.30 oil objective. Image deconvolution was carried out on each channel individually using the Huygens Professional (Scientific Volume Imaging) software with a back-projected pinhole of half the emission wavelength in namometers, a theoretical Point Spread Function, automatic background estimation, five iterations, a signal-to-noise ratio of 20, a Quality threshold of 0.05, optimized iteration mode, and an automatic brick layout. The separate deconvolved channels were then combined as an RGB tiff using Fiji (Schindelin *et al.* 2012).

### Fluorescence quantification

For the comparison between old and new reporters we acquired confocal stacks using two different 561-nm laser power settings (low 2% and high 10%) with gain (600) and pinhole (60.1 μm, 1.42 AU) remaining constant. Images acquired at the low setting were optimal for nonsaturated images of the new reporters and images acquired at the high setting were optimal for the old reporters so that we had a stack that could be segmented for quantification and then the data from the low stacks were quantified (see below). Confocal .lsm files were then converted to .nrrd files using Fiji. Using Amira 6.0.1 (FEI, Thermo Fisher Scientific) a .nrrd stack, for each brain to be quantified, was opened (high versions for the old reported and low versions for the new reporters) and a median filter of three iterations was applied. Using the Segmentation Editor in Amira 6.0.1, two materials were assigned to the median filtered stack for each brain: (1) for quantifying signal a three-dimensional ROIs surrounding the axonal terminals of Mz19-Gal4 PNs in the lateral horn and (2) for background correction a three-dimensional region ventral to the axonal terminals of Mz19-Gal4 PNs in the lateral horn. The intensity and background correction calculations were performed in R (R Core Development Team 2016) and detailed in File S2. Briefly, for comparison of the old and new CLIPf reporters we used the average intensity in the LH of the old reporters as baseline and then divided the quantified intensity of the new reporter by the average for the old reporters to give a fold change (*e.g.*, for the comparison of new 4xCLIPf in attP40 with the old version of the CLIPf reporters: the intensity value of 4xCLIPf in attP40 was divided by the average of the intensities calculated for both insertions of the old version CLIPf reporters, see File S2 for details of the calculations). For new *vs.* old comparisons of the Halo reporters, we calculated percentage change as this was a more meaningful comparison (see File S2 for details of the calculations).

### Data availability

All data necessary for confirming the conclusions presented in the article are represented fully within the article. All fly strains and plasmids are available upon request. Sequence data for all plasmids will be made available at GenBank and the accession numbers listed in Table 1. Code used to quantify fluorescence intensities is provided in File S2.

## Results

### New CLIPf and SNAPf reporters with increased sensitivity

The first generation of chemical labeling reporters achieved rapid staining times, shortening protocols from over 100 hr to <1 hr for whole-mount *Drosophila* brains (Kohl *et al.* 2014). Despite this dramatic improvement in staining speed, signal strength is lower than antibody staining of reporter proteins. This is likely due to the nonamplifying nature of chemical labeling: one molecule of tag covalently binds one substrate molecule fused to one molecule of fluorophore. This linearity can be beneficial when quantifying signal intensity. In contrast, with immunofluorescence one target can be bound by more than one primary antibody which is then recognized by several secondary antibody molecules, each conjugated to multiple fluorophores leading to substantial signal amplification. This lower sensitivity is evident when comparing the signal from several Gal4 lines [Rubin collection, Janelia Research Campus (Jenett *et al.* 2012)] driving GFP or first-generation CLIPf and SNAPf reporters (Figures S1a and S2a in File S1). To bridge this gap and extend the use of chemical labeling to most Gal4 driver lines, weak and strong, we designed a new generation of reporters with greatly increased sensitivity. These reporters differ from the original ones in two ways: first, they have a short 5′ UTR (AcNPV) and the 3′ UTR from the *A. californica nucleopolyhedrovirus* P10 gene – these modifications have been shown to increase translational efficiency by >20 times (Pfeiffer *et al.* 2012); and second, they are tetramerized to increase reporter signal up to fourfold (Shearin *et al.* 2014) (Figure 1A). We generated transgenic fly lines by inserting these new 4xCLIPf and 4xSNAPf reporters into the well-characterized attP40 and VK00005 phiC31 landing sites on the second and third chromosomes, respectively (Table S1 in File S1).

We tested these new transgenes and compared them to the first-generation reporters using the sparse line Mz19-Gal4, a driver of medium strength that expresses in about 12 olfactory projection neurons innervating three adjacent olfactory glomeruli and a group of neurons with processes near the mushroom bodies. When driven by Mz19-Gal4, all reporters produced the expected labeling pattern. In comparison, the first-generation tags were barely visible when imaged under conditions that produced strong signal with the new reporters (Figure 1B). To quantify the increase in signal strength we measured intensity in the axonal terminals of projection neurons in the lateral horn (green dotted area in Figure 1D, see *Materials and Methods*). Using the average between UAS-CD4::CLIPf on the second and third chromosomes as baseline, the new UAS-myr::4xCLIPf reporters are 64 (attP40) and 24 (VK00005) times brighter. In the case of SNAP, the new UAS-myr::4xSNAPf reporters are 7 (attP40) and 10 (VK00005) times brighter than the average between the first generation UAS-myr::SNAPf in attP2 and attP40. While CLIPf and SNAPf substrates use different fluorophores and have different labeling sensitivities, complicating precise quantitative comparisons, the new CLIPf and SNAPf reporters produced qualitatively similar fluorescence intensities. To extend these results to other driver lines we used a number of Gal4 *P* element and enhancer fusion insertions of varying strengths to drive the new reporters (weakest to strongest: GMR-50A02-Gal4, GMR-59F02-Gal4, and GMR-54F05-Gal4). Qualitatively these stainings recapitulated the Mz19-Gal4 results with the new reporters showing large increases in brightness (Figures S1 and S2 in File S1). These results indicate that the new reporters are suitable for labeling most, if not all, Gal4 driver lines that show expression after immunostaining.

### LexA-responsive reporters

Dissecting the function of neuronal components in a circuit often requires labeling more than one cell population with different reporters that respond to orthogonal drivers such as Gal4 and LexA. To increase the flexibility of the chemical labeling platform we made LexA-responsive tetramerized CLIPf and SNAPf reporters and inserted them in attP40 and VK00005 (Table S1 in File S1). We tested these reporters using the weak driver line GH146-LexA::GAD. We found that LexAop2-myr::4xCLIPf and LexAop2-myr::4xSNAPf reporters inserted in both chromosomal locations produced strong labeling (Figure 1C and Figure S2c in File S1). Since new LexA drivers are now routinely made with the strong p65 transactivation domain rather than the weaker GAD domain, this result suggests our new reporters will be useful for most LexA driver lines. Finally, we show how these new reagents can be used for visualizing different cell populations by labeling olfactory sensory neurons (Orco-LexA::VP16) and a subset of their postsynaptic projection neurons (Mz19-Gal4) in the same brain (Figure 1D). While we imaged this brain using a confocal microscope (following the Nyquist criterion and subsequent deconvolution, see *Materials and Methods*), superresolution microscopy techniques, such as stimulated emission depletion (STED), could also be used, when available for thick tissue specimens, to increase resolution.

### New Halo tag reporters with improved membrane localization and signal strength

**Figure 2:**
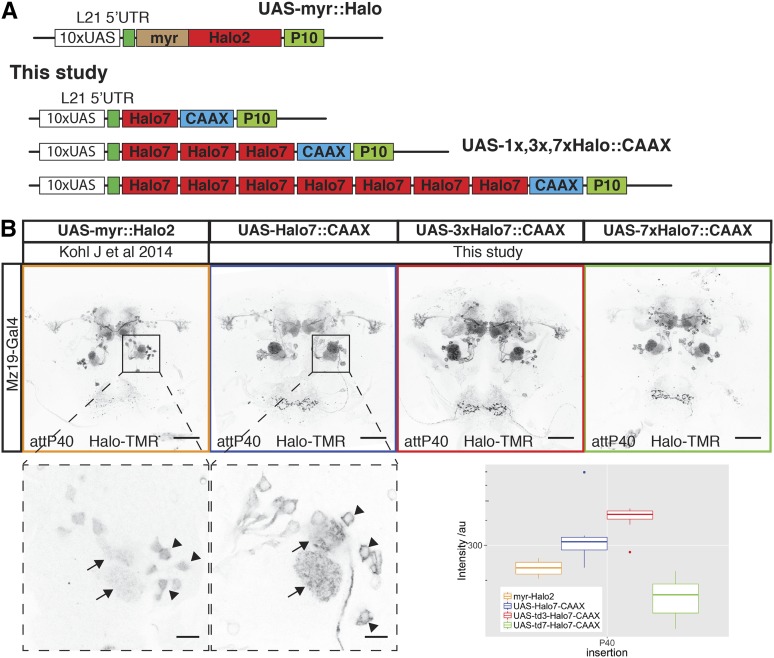
New Halo reporters with improved sensitivity and localization. (A) Schematic of Halo reporters from Kohl *et al.* (2014) and the new reporters from this study. (B) Labeling of Mz19-Gal4-positive neurons using the old myr::Halo2 and new Halo7::CAAX reporters. All images were aquired using the same microscope settings. Bottom panels are high-magnification single-slice images showing differences in reporter localization in the cell bodies (arrowheads) of olfactory projection neurons. Arrows indicate signal in glomeruli. The boxplot shows the quantification of fluorescence intensity of the axonal terminals of PNs in the lateral horn (arbitrary units). Boxplot *n* numbers were: myr::Halo2 *n* = 7, UAS-Halo7::CAAX-P40 *n* = 7, UAS-3xHalo7::CAAX *n* = 8, and UAS-7xHalo7::CAAX *n* = 8. Bars in full brain images are 50 μm and higher magnification images of cell bodies are 10 μm.

Our first-generation Halo tag reporters already incorporated the 5′ and 3′ translational enhancers L21 and P10 (Figure 2A) and were inserted into PhiC31 landing sites that support strong expression (attP40 and attP2). While this tag produced the brightest signal among the first generation of chemical reporters, we noticed an unexpected accumulation of the tag in the cell nucleus and reduced signal in axons (Figure 2B) suggesting suboptimal cellular localization. Intriguingly, 4xCLIPf and 4xSNAPf tags use the same myristoylation signal as Halo (first 90 amino acids from the *Drosophila* Src protein) but are excluded from the nucleus, displaying the expected membrane localization. In order to improve cellular localization, we replaced the N-terminal myristoylation with a C-terminal CAAX membrane targeting signal (Choy *et al.* 1999). In addition, we made several reporters with one, three, or seven tandem fusion-tags of Halo with the aim of increasing labeling efficiency (Figure 2A). The new constructs use Halo version 7 (Halo7) which is reported to show increased expression, stability, and substrate-binding kinetics over version 2 (Halo2) (Encell *et al.* 2012). We made transgenic flies with insertions in attP40, VK00005, and VK00027 (Table S1 in File S1).

We compared cellular localization and signal intensities from the first and new generation of Halo tags in the same way as for CLIPf and SNAPf. Nuclear signal is greatly reduced in the new CAAX reporters when compared to the myristoylated ones (see higher magnification images from the first two panels of Figure 2B). In addition, we measured modest increases in signal strength with the new monomeric and trimeric reporters (53 and 78% brighter, respectively, Figure 2B, boxplot). Surprisingly, the heptamer is 28% less bright than the old reporter, possibly due to increased instability or impaired trafficking (Figure 2B, boxplot).

### Chemical tags in peripheral sensory organs

**Figure 3:**
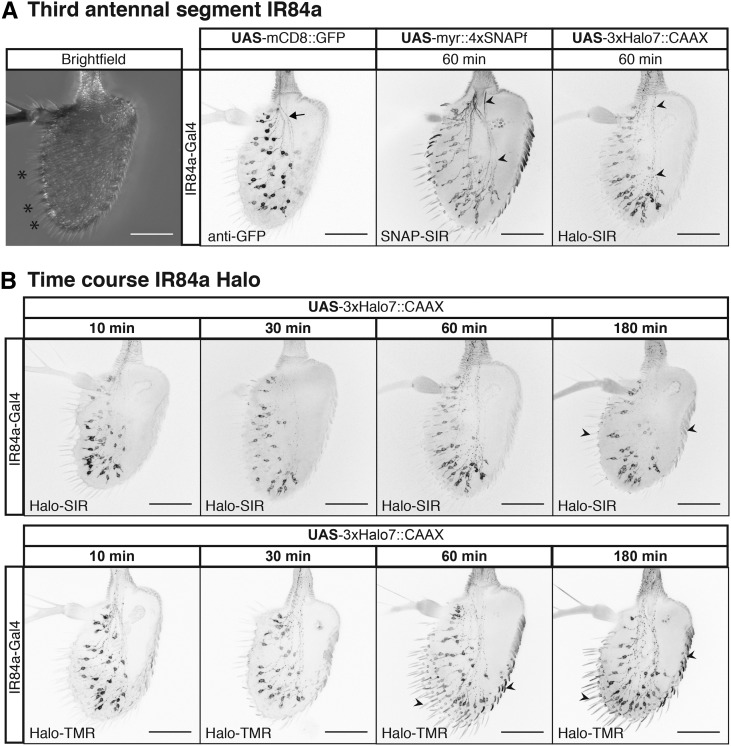
Chemical tags in peripheral sensory organs. (A) Left-most image, bright field image of the antennae, sensilla are marked with asterisks. Chemical labeling of Ionotropic Receptor 84a (IR84a) expressing sensory neurons. Comparison between GFP immunostaining and SNAP-SiR and Halo-SiR chemical labeling; arrow and arrowheads highlight stronger axonal chemical labeling. (B) Incubation time series for far red Halo-SiR (top row) and red Halo-TMR (bottom row) dyes. All panels shows partial projections of confocal stacks that exclude the cuticle. Bars, 50 μm.

We wanted to explore the performance of chemical labeling in tissues other than the brain, where differences in extracellular matrix or other cellular barriers may have a negative impact on labeling. To accomplish this we stained sensory neurons in whole-mount third antennal segments. This tissue is typically regarded as hard to stain in part because it is surrounded by cuticle, in contrast to brains which are dissected out of the head capsule before staining. While immunolabeling can work, as for brains, the optimized protocol spans up to a week (Saina and Benton 2013). Using GAL4 driver lines that label sensory neurons [Ionotropic Receptor 84a (IR84a) and Odorant Receptor 22a (OR22a)], we expressed the new 4xSNAPf and 3xHalo7 reporter lines in the antennae (Figure 3 and Figure S3 in File S1). While reporters produced signal in the expected cells in all cases, shorter labeling incubations produce lower background, especially in the cuticle (Figure 3B, arrowheads). The SNAPf label also resulted in more uniform labeling of the axons and soma when compared to a mCD8::GFP reporter (Figure 3A, arrowheads *vs.* arrows). In contrast to immunostaining, chemical labeling reagents penetrate rapidly as demonstrated by the signal being as strong after 10 min as it is after 3 hr (Figure 3B). In addition, chemical labeling in the antennae, as in the brain (Kohl *et al.* 2014), can be combined with immunolabeling, in this case of the OR22a receptor (Figure S3 in File S1).

### Conditional reporters for sparsening labeling

A fundamental step in studying complex neural circuits is to break them down into smaller components by visualizing the morphology of single or small clusters of neurons. Gal4 and LexA lines often have overlapping processes which cannot be resolved by light microscopy. In these cases, further labeling refinements, using a number of genetic strategies, are required (Jefferis and Livet 2012). We extended the applicability of chemical labeling to these situations by developing reagents to: (a) limit the number of labeled cells or (b) increase the combinatorial number of fluorophores available for each labeled neuron.

**Figure 4:**
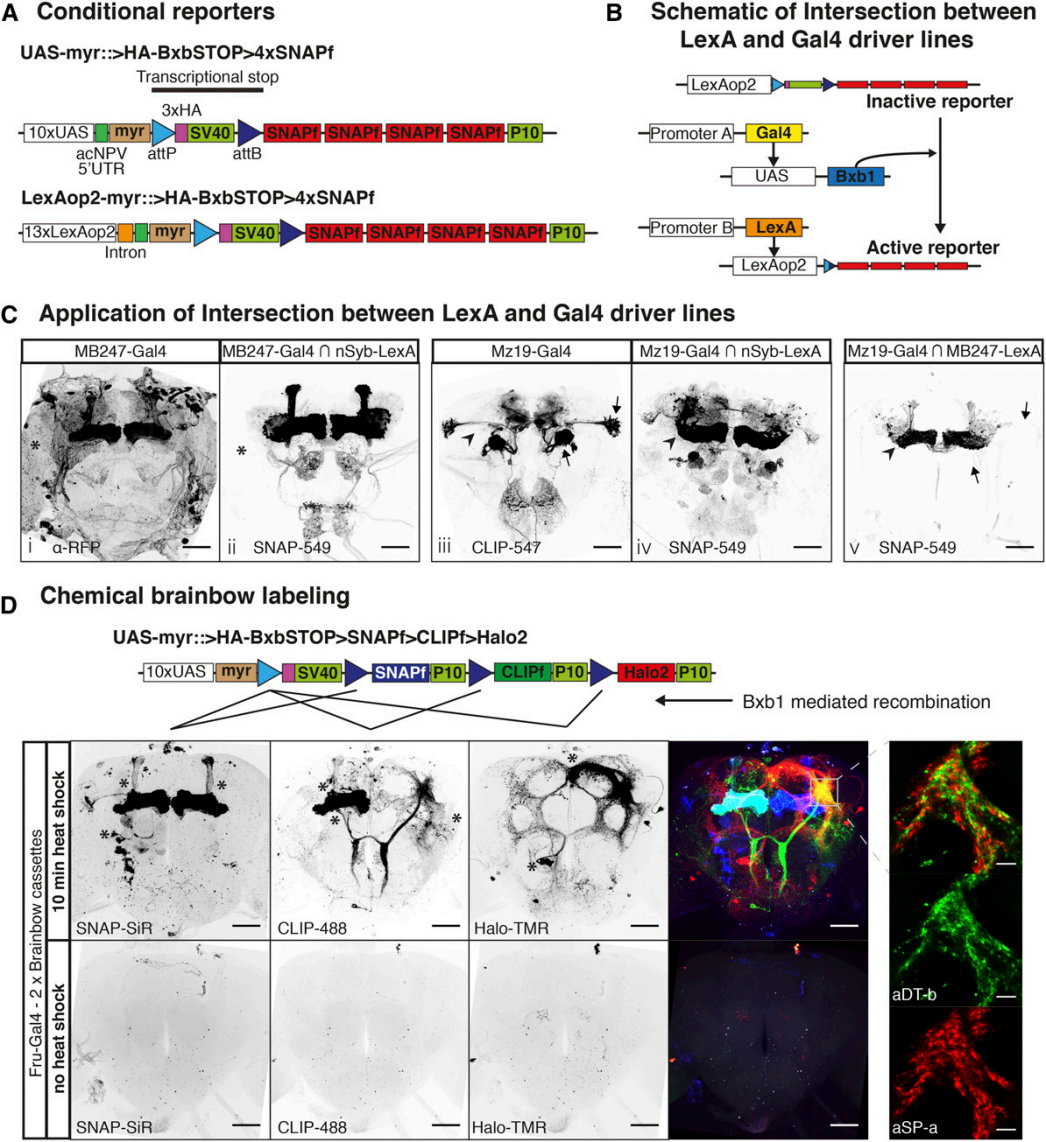
Sparsening expression using conditional chemical reporters. (A) Schematic of new conditional reporters. The HA tag present in the stop cassette can reveal the expression of the inactive reporter (not shown). (B) Schematic showing the genetic approach to intersect LexA and Gal4 in C. (C) Images i and iii show confocal projections of Gal4 lines driving regular reporters. Images ii and iv show confocal projections of Gal4 lines intersected with the panneuronal nSyb-LexA::P65 using the scheme from B. Image v shows a confocal projection of the intersections between the sparse lines Mz19-Gal4 and MB247-LexA::VP16. Asterisks indicate glial staining in image i and lack of in ii. Arrowheads indicate lack of Mushroom Body (MB) staining in image iii and presence of staining in iv. Arrows show loss of Projection Neuron signal and arrowhead shows increase in MB signal in image v relative to image iii. (D) Heatshock activation of Brainbow cassettes during early development label neuroblast clones of *fruitless* positive neurons. Bottom panels show the Brainbow cassettes are silent when no heatshock is applied. Asterisks indicate the cell bodies from neuroblast clones. Panels on the right: high magnification single confocal slice showing the close apposition between processes from the two sexually dimorphic clones aSP-a and aDT-b. Bars for full brain images are 50 μm and bars for higher magnifications are 10 μm.

To limit the number of labeled cells we designed an inactive reporter with a transcriptional stop cassette upstream of the coding region for 4xSNAPf. This reporter can be activated upon removal of the stop cassette by the DNA recombinase Bxb1 (Figure 4A). We chose Bxb1 from mycobacteriophage (Huang *et al.* 2011) as it is orthogonal to recombinases commonly used in *Drosophila*, including Flp and PhiC31. Another advantage is its irreversibility as it recombines attP and attB sites to generate new attL and attR sites which are no longer substrates. We generated lines that express Bxb1 in three different ways: (a) stochastically, using a heat shock inducible promoter (hs-Bxb1, Figures S4 to S12 in File S1); (b) by driving its expression with Gal4 (UAS-Bxb1, Figure 4B); and (c) by using a combination of Gal4 and Flp DNA recombinase (UAS > FlpSTOP > Bxb1, Table S1 in File S1).

As a proof of principle, we used the conditional reporters in three experiments to intersect the expression of Gal4 and LexA drivers. The schematic in Figure 4B shows the logic of the experiment: MB247-Gal4 or Mz19-Gal4 drives expression of UAS-Bxb1 to activate the conditional reporter LexAop2-myr::>BxbSTOP > 4xSNAP; the activated reporter is then driven by MB247-LexA::VP16 or the panneuronal nSyb-LexA::p65. In the first experiment, MB247-Gal4 ⋂ nSyb-LexA::P65, the result is very similar to that of a regular reporter with the exception of the lack of strong glial staining, normally present in MB247-Gal4, due to the reporter being driven by the neuronal specific nSyb-LexA::p65 (compare asterisks in Figure 4C, i and ii). On the other hand, the second experiment shows that Mz19-Gal4 ⋂ nSyb-LexA::P65 expression is considerably broader than that of the regular reporter including labeling in the mushroom bodies (compare arrowheads in Figure 4C, iii and iv). Mz19-Gal4 ⋂ nSyb-LexA::P65 reflects two interesting properties of this approach: first, it captures and immortalizes developmental expression; and second, weakly expressing cells, previously undetectable with a regular reporter, could drive Bxb1-mediated recombination allowing strong reporter expression driven by nSyb-LexA::P65 (arrowheads in Figure 4C, iii and iv). In the third experiment, we used Mz19-Gal4 to activate the reporter and MB247-LexA::VP16 to drive it; as one would predict from the previous two experiments, this intersection labels a modest number of mushroom body Kenyon cells (arrowhead in Figure 4C v) while expression in the PNs is absent (compare arrows in Figure 4C, iii and v).

The second strategy for resolving overlapping processes is multiplexing the label. The approach we took is based on the Brainbow technique (Livet *et al.* 2007; Hadjieconomou *et al.* 2011; Hampel *et al.* 2011) using the tags CLIPf, SNAPf, and Halo2 (Figure 4D). Our reporter incorporates translational enhancers without multimerization. We used Bxb1 to activate the cassette as for our single tag conditional reporters. Because Bxb1 recombination is irreversible, the cassette requires fewer recombination sites than previous Brainbow reporters. Upon expression of the recombinase, the single attP site recombines with one of the three attB sites removing the intervening DNA and irreversibly selecting one of the three tags for expression (see schematic in Figure 4D). We made fly lines with the Brainbow cassette inserted into attP2 and VK00005 (Table S1 in File S1).

We tested the new cassettes by labeling subsets of neurons that express the male-specific form of the Fruitless protein (FruM). By activating the Brainbow cassette immediately after larval hatching we aimed to create groups of labeled cells of the same developmental origin (neuroblast clones, see *Materials and Methods*). Our pilot experiment showed that both transgenes are efficiently activated producing the expected *fruitless* positive neuroblast clones [compare Figure 4D with Cachero *et al.* (2010)]. We found that the three chemical tags were activated in a similar number of neuroblast clones (marked with asterisks in Figure 4D: three clones for SNAPf, three for CLIPf 3, and two for Halo2). The presence of both Brainbow cassettes can be seen in the mushroom body clone on the fly’s right side where both CLIPf and SNAPf tags were activated, labeling the resulting clone in cyan. Resolving several clones in a single brain has the advantage of requiring fewer samples to describe the anatomy of a neuronal population. Furthermore, it enables researchers to examine the overlap between clones within the same brain rather than using image registration and *post hoc* comparisons of clones from multiple brains. For instance, this enabled examination of the close apposition of processes from aSP-a and aDT-b clones in the male enlarged region of the brain (Figure 4D, high magnification insets).

### Subcellular reporters

Finally, we generated reporters for other cellular compartments, both in the nervous system and elsewhere.

**Figure 5:**
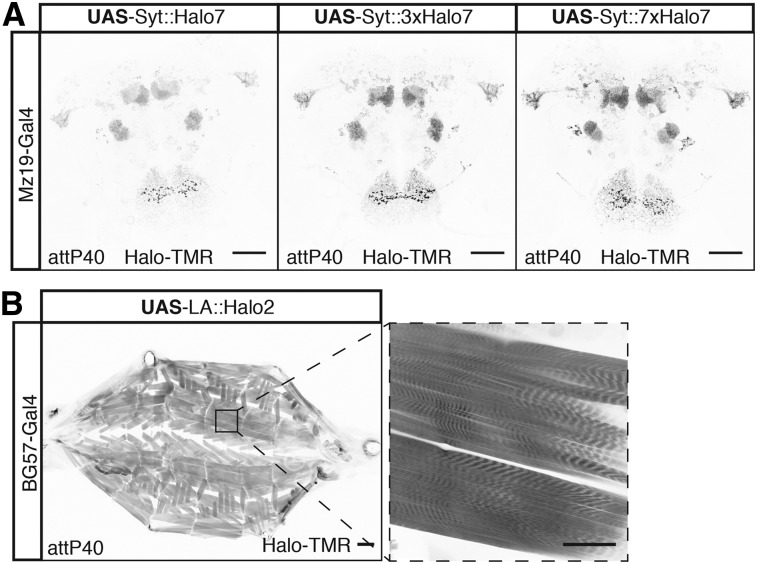
New subcellular Halo reporters. (A) Labeling the synaptic terminals of Mz19-Gal4-positive neurons using Halo7 reporters fused to Synaptotagmin. (B) Labeling of muscle actin in the larva using a fusion between Halo2 and LifeAct peptide. Bars in full brain images and higher magnification of muscle fiber are 50 μm and full larva are 200 μm.

Synapses are the key sites of information transfer in neuronal circuits. In order to label them, we made UAS reporters where one, three, or seven copies of the Halo7 tag are fused to the presynaptic protein Synaptotagmin (Syt, Table S1 in File S1). When driven by Mz19-Gal4 all three Syt::Halo7 synaptic markers produced strong labeling in areas known to have presynapses with minimal presence in regions devoid of them (compare Figure 2B and Figure 5A). The gradation in signal strength going from monomer to heptamer makes these reporters useful for labeling synapses using drivers ranging from weak to strong.

Next, we made a reporter for fast and sensitive labeling of actin filaments by fusing a peptide, LifeAct (LA), that binds actin filaments to Halo2 (Table S1 in File S1) (Riedl *et al.* 2008). As a proof of principle, we expressed the reporter using the pan-muscular driver BG57-Gal4. These larvae are viable despite widespread expression of LA::Halo2, indicating the reporter is not overtly toxic. The staining of body wall muscles in third instar larvae revealed the expected expression pattern with stripes of muscle actin bundles clearly visible (Figure 5B).

## Discussion

In this study we introduce a second generation of chemical tags that achieve substantial improvements in sensitivity and versatility over the first generation. Most applications where tag immunostaining is used can benefit from super fast and highly sensitive chemical labeling and the new reagents are ideally suited for medium to high throughput applications such as anatomical screens of driver lines or assessment of RNAi screen phenotypes.

The introduction of LexAop2 and conditional reporters opens the possibility to a larger set of experiments than was possible with first-generation reagents. For instance, combining UAS and LexAop2 reporters will allow superresolution microscopy to resolve potential contacts between different neuronal populations. The Brainbow cassette can be used in large anatomical screens enabling rapid characterization of complex driver lines by labeling multiple clones in the same brain (Livet *et al.* 2007; Hadjieconomou *et al.* 2011; Hampel *et al.* 2011). Besides the increase in speed, this allows imaging different neuronal populations in the same brain offering a powerful insight into their potential connectivity. Our conditional reporters can be used to capture developmental expression; these could be exploited for a systematic study of neuronal fate during metamorphosis. While we validated our reagents in the antennae, it is likely that chemical labeling will work in most other tissues. Beyond the field of neuroscience, the chemical actin reporter will be a useful alternative to the widely used but highly toxic phalloidin staining, particularly in those applications where genetically targeting to specific muscles could be an advantage. A second advantage is the irreversible nature of the chemical staining, while phalloidin stainings fade with time. Lastly, it could be used for *in vivo* imaging when combined with cell-permeable substrates.

The improvements in signal strength achieved by the new reagents derive from their higher expression levels. For experiments where an even stronger signal is needed, more than one transgene could be used. In the case of the Brainbow cassettes, we are currently multimerizing the tags to obtain a higher signal-to-noise ratio. Another possibility would be developing brighter ligands, for instance by conjugating multiple fluorophores per ligand molecule. The collection of reagents presented here is by no means exhaustive and further additions to this toolkit could include generation of reporters to harness the QUAS system (Potter *et al.* 2010) and expansion of the multimerized chemical tags to target subcellular compartments and organelles; for example, axons, dendrites, microtubules, and mitochondria. The recent development of CRISPR opens an exciting avenue for incorporating these chemical tags into endogenous proteins (Gratz *et al.* 2013). Another application could be their use as protein tags in bacterial artificial chromosomes or fosmids.

While the new chemical tags were successful in producing strong labeling of all Gal4 and LexA lines tested, a new comparison between chemical labeling and “spaghetti monster” Fluorescent Protein (smFP) immunolabeling (Viswanathan *et al.* 2015) found that the latter still yields better signal-to-noise ratio than a single copy multimerized chemical tag (G. Meissner, personal communication). This is unsurprising as the smFPs are one of the most optimized tags available for immunostaining with 10–15 copies of their epitope tags, which are then subjected to a highly refined, but long (>10 days), staining protocol. Therefore, in our view the significant increase in speed and reproducibility derived from the simple chemical labeling protocol, coupled with strong signal, make it an attractive option for most applications.

In conclusion, the new reagents generated in this study significantly extend the experimental reach of chemical labeling to most forms of genetic labeling scenarios in *Drosophila*. This should significantly increase its use by the research community. We hope that this will also encourage non-*Drosophila* researchers to expand and optimize the use of chemical labeling in other model organisms.

## Supplementary Material

Supplemental material is available online at www.genetics.org/lookup/suppl/doi:10.1534/genetics.116.199281/-/DC1.
