# Supplementary material for: Second-Generation Drosophila Chemical Tags: Sensitivity, Versatility, and Speed
Source: Genetics. 2017 Feb 14;205(4):1399–408. doi: 10.1534/genetics.116.199281 (PMC5378102; doi:10.1534/genetics.116.199281)
Supplement: Supplementary file 1 [file 1399FileS1.pdf]

Supplemental Information

Materials and Methods

Labeling of tissues with one or two tags was performed as in Kohl et al. (2014). For Brainbow and Antennal labeling protocols see below.

Triple labeling of Chemical Brainbow Drosophila Brains

We made a double cassette reporter by combing both Brainbow insertions with hs-Bxb1 and *fruitless*-Gal4; a knock in insertion of Gal4 into the P1 promoter of the Fru locus (Stockinger et al., 2005). First we screened several hs-Bxb1 insertions and identified one that produces minimal background activation of the reporters at 25° (that is the line that gave fewest labelled cells with no heat shock). Next, newly hatched larvae were heat-shocked for 10 minutes at 37° and allowed to develop into adults. Flies were then processed as follows:

- All steps were carried out at room temperature unless stated differently.
- Brains were dissected in ice-cold 0.1 M phosphate buffer (PB: 0.032 M NaH_2PO_4, 0.068 M NaH_2PO_4).
- Fixed in 4 % paraformaldehyde (PFA) (in 0.1 M PB) for 30 min in a glass-well plate on an orbital shaker
- Transferred to 1.5 ml tube
- Permeabilized by incubation in 1 ml of PBS-T (phosphate buffered saline + 0.3 % Triton X-100) (2 x 15 min) on rotating wheel
- Incubated with SNAP-Cell 647-SiR (NEB: S9102S) substrate at a final concentration of 1 µM in PBS on rotating wheel for 30 min

- Added CLIP-Surface 488 (NEB: S9232S) and HaloTag TMR (Promega: G8252) at a final concentration of 1 µM to the tube with brains/SNAP-Cell 647-SiR solution

- Incubated on rotating wheel for a further 30 mins

- Washed with PBS-T (2 × 15 min)

- PBS-T was removed as completely as possible and 100 µl of Vectashield (or other) mounting medium added. We observed that subsequently transferring brains into a fresh 100 µl aliquot of Vectashield results in more homogeneous signal along the z axis of the image

- After labeling brains were then mounted on charged slides and imaged

Chemical labeling of Drosophila Antennal Segments

- All steps were carried out at room temperature unless stated differently.

- Antennae were harvested in liquid nitrogen Saina and Benton (2013)

- Fixed in 4% PFA, 3% Triton, 1xPBS (180 min)

- Washed in 3% Triton, 1xPBS (2 x 5 min)

- Washed in 0.1% Triton, 1xPBS (2 x 5 min)

- Incubated in 5 µM Halo-SIR (10 min)

- Washed in 0.1% Triton, 1xPBS (2 x 10min)

- Washed in 0.1% Triton, 1xPBS (2 x 5min)

- After labeling antennae were mounted in Vectashield and imaged

Chemical and Antibody co-labeling Drosophila Antennal Segments

- All steps were carried out at room temperature unless stated differently.
- Antennae were harvested in liquid nitrogen
- Fixed in 4% PFA, 3% Triton, 1xPBS (180 min)
- Washed in 3% Triton, 1xPBS (2 x 10min)
- Washed in 0.1% Triton, 1xPBS (2 x 10min)
- Incubated in 5 µM Halo-SIR (10 min)
- Washed in 0.1% Triton, 1xPBS (2 x 10min)
- Blocked in 5% goat serum, 0.1%Triton, 1xPBS (60 min)
- Incubated with primary antibody in in 5% goat serum, 0.1%Triton, 1xPBS (overnight at 4°)
- Washed in 0.1% Triton, 1xPBS (6 x 15min)
- Blocked in 5% goat serum, 0.1%Triton, 1xPBS (60 min)
- Incubated with secondary antibody in in 5% goat serum, 0.1%Triton, 1xPBS (overnight at 4°)
- Washed in 0.1% Triton, 1xPBS (6 x 15min)
- After labeling antennae were mounted in Vectashield and imaged

Tables

Tag	Promoter	Insertion site	Conditional	Transgene	Figure
CLIP	UAS	VK00005	-	UAS-myr::4xCLIPf	1, S1
		attP40	-	UAS-myr::4xCLIPf	1, S1
	LexAop2	VK00005	-	LexAop2-myr::4xCLIPf	S2
		attP40	-	LexAop2-myr::4xCLIPf	1
SNAP	UAS	VK00005	-	UAS-myr::4xSNAPf	1, S2
		attP40	-	UAS-myr::4xSNAPf	1, S2
	LexAop2	VK00005	-	LexAop2-myr::4xSNAPf	S2
		attP40	-	LexAop2-myr::4xSNAPf	1
	UAS	VK00005	yes	UAS-myr::>BxbSTOP>-4xSNAPf	-
	LexAop2	VK00018	yes	LexAop2-myr::>BxbSTOP>-4xSNAPf	4
Halo	UAS	attP40	-	UAS-Halo7::CAAX	2
		attP40	-	UAS-3xHalo7::CAAX	2,3
		attP40	-	UAS-7xHalo7::CAAX	2
		attP40	-	UAS-Syt::Halo7	5
		attP40	-	UAS-Syt::3xHalo7	5
		attP40	-	UAS-Syt::7xHalo7	5
		attP40	-	UAS-LA-Halo2	5
Bxb1	heat shock	attP18	-	HeatShock-Bxb1	-
	heat shock	attP40	-	HeatShock-Bxb1	-
	heat shock	P element	-	HeatShock-Bxb1	4
	UAS	VK00027	yes	UAS->FlpSTOP>Bxb1	-
	UAS	VK00027	-	UAS->Bxb1	4
SNAPf CLIPf Halo	UAS	attP2	yes	UAS-ChemicalBrainbow	-
		VK00005	yes	UAS-ChemicalBrainbow	-
		attP2, VK00005	yes	UAS-2xChemicalBrainbow	4

Table S1: **Transgenic flies generated in this study.** These stocks have been deposited at Bloomington Stock Center and can be found here http://flystocks.bio.indiana.edu/Browse/misc-browse/chemtag.php

Genotype	Purpose
w ; LexAop2-myr::>BxbSTOP >-4xSNAPf(VK00018) / CyO ;UAS->Bxb1(VK00027) / TM6	When crossed to flies with Gal4 and LexA drivers it labels their intersection with SNAP. See Figure 4c.v.
w ; nSyb-LexA::P65 LexAop2-myr::>BxbSTOP >-4xSNAPf(VK00018) / CyO ;UAS->Bxb1(VK00027) / TM6	When crossed to flies with a Gal4 driver it labels and "immortalises" SNAP expression in neurons. See Figure 4c.ii and iv.

Table S2: **Stocks for conditional SNAP labeling.** These stocks have been deposited at Bloomington Stock Center and can be found here http://flystocks.bio.indiana.edu/Browse/misc-browse/chemtag.php

Figure	Genotype in figure	Full Genotype
1b	Mz19-Gal4 > UAS-CD4::CLIFf, 2nd chr.	; Mz19-Gal4 / UAS-CD4::CLIPf ; + / Ki
1b	Mz19-Gal4 > UAS-myr::4xCLIPf, attP40	; MZ19-Gal4 / UAS-myr::4xCLIPf in attP40 ;
1b	Mz19-Gal4 > UAS-myr::4xCLIPf, VK00005	; Mz19-Gal4 / CyO;UAS-myr::4xCLIPf in VK00005 / +
1b	Mz19-Gal4 > UAS-myr::SNAPf, attP40	; MZ19-Gal4 / UAS-myr::SNAPf in attP40-5 ; + / Ki
1b	Mz19-Gal4 > UAS-myr::4xSNAPf, attP40	; MZ19-Gal4 / UAS-myr::4xSNAPf in attP40 ; + / MKRS
1b	Mz19-Gal4 > UAS-myr::4xSNAPf, VK00005	; MZ19-Gal4 / CyO;UAS-myr::4xSNAPf in VK00005 / +
1c	GH146-LexA > LexAop2-myr::4xCLIPf, attP40	hsFLP* ; GH146-LexA / LexAop2-myr::4xCLIPf in attP40 ; MB247-Gal4*,QUAS-mtdTomato*/MKRS
1c	GH146-LexA > LexAop2-myr::4xSNAPf, attP40	hsFLP* ; GH146-LexA / LexAop2-myr::4xSNAPf in attP40 ; MB247-Gal4*, QUAS-mtdTomato*/MKRS
1d	Mz19-Gal4 > UAS-myr::4xSNAPf, LexAop2-myr::4xCLIPf	; MZ19-Gal4 / LexAop2-myr::4xCLIPf in attP40 ; OrcoLexAVP16 / UAS-myr::4xSNAPf in VK00005
2b	Mz19-Gal4 > UAS-myr::Halo2, attP40	; MZ19-Gal4, UAS-myr::Halo2 in attP40/CyO ; TM6B / +
2b	Mz19-Gal4 > UAS-Halo7::CAAX, attP40	; MZ19-Gal4 / UAS-Halo7::CAAX in attP40 ;
2b	Mz19-Gal4 > UAS-3xHalo7::CAAX, attP40	; MZ19-Gal4 / UAS-3xHalo7::CAAX in attP40 ;
2b	Mz19-Gal4 > UAS-7xHalo7::CAAX, attP40	; MZ19-Gal4 / UAS-7xHalo7::CAAX in attP40 ;
3a	IR84a-Gal4 > UAS-mCD8::GFP	; UAS-mCD8::GFP ; IR84a-Gal4
3a	IR84a-Gal4 > UAS-myr::4xSNAPf	; UAS-myr::4xSNAPf in attP40 ; IR84a-Gal4
3a, b	IR84a-Gal4 > UAS-3xHalo7::CAAX	; UAS-3xHalo7::CAAX in attP40 ; IR84a-Gal4
4c.i	MB247-Gal4	LexAop2-mCD8::GFP*, UAS-mCD8::RFP/+ ; ; MB247-Gal4 / +
4c.ii	MB247-Gal4 $\bigcap$ nSyb-LexA	; LexAop2-myr::>HA-BxbSTOP>4xSNAPf / nSyb-LexAP65 ; UAS>Bxb1 / MB247-Gal4, QUAS-mtdTomato*
4c.iii	Mz19-Gal4	; MZ19-Gal4 / UAS-myr::4xCLIPf in attP40 ;
4c.iv	Mz19-Gal4 $\bigcap$ nSyb-LexA	; nSyb-LexAP65 LexAop2-myr::>HA-BxbSTOP>4xSNAPf / MZ19-Gal4 ; UAS>Bxb1 / +

Figure	Genotype in figure	Full Genotype
4c.v	Mz19-Gal4 $\bigcap$ MB247-LexA	; Mz19-Gal4 / LexAop2-myr::>HA-BxbSTOP>4xSNAPf ; MB247-LexA / UAS-Bxb1
4d	Fru-Gal4 > 2 x Brainbow cassettes	; hsBxb1 / CyO ; Fru-Gal4 / UAS-myr::>HA-BxbSTOP>SNAPf>CLIPf>Halo2 in attP2, UAS-myr::>HA-BxbSTOP>SNAPf>CLIPf>Halo2 in VK00005
5a	Mz19-Gal4 > UAS-Syt::Halo7	Mz19Gal4 / UAS-Syt::Halo7 in attP40
5a	Mz19-Gal4 > UAS-3xSyt::Halo7	Mz19Gal4 / UAS-3xSyt::Halo7 in attP40
5a	Mz19-Gal4 > UAS-7xSyt::Halo7	Mz19Gal4 / UAS-7xSyt::Halo7 in attP40
5b	BG57-Gal4 > UAS-LA::Halo2	UAS-Dicer2 ; UAS-LA::Halo2 in attP40 / + ; BG57Gal4 / +
S1a	Mz19-Gal4 > UAS-CD4::CLIPf on 2nd	; Mz19-Gal4 / UAS-CD4::CLIPf ;
S1a	50A02-Gal4 > UAS-CD4::CLIPf on 2nd	; UAS-CD4::CLIPf / + ; 50A02-Gal4 / +
S1a	54F05-Gal4 > UAS-CD4::CLIPf on 2nd	; UAS-CD4::CLIPf / + ; 54F05-Gal4 / +
S1a	59F02-Gal4 > UAS-CD4::CLIPf on 2nd	; UAS-CD4::CLIPf / + ; 59F02-Gal4 / +
S1a	Mz19-Gal4 > UAS-CD4::CLIPf on 3rd	; Mz19-Gal4 / + ; UAS-CD4::CLIPf / + ;
S1a	50A02-Gal4 > UAS-CD4::CLIPf on 3rd	; ; UAS-CD4::CLIPf / 50A02-Gal4
S1a	54F05-Gal4 > UAS-CD4::CLIPf on 3rd	; ; UAS-CD4::CLIPf / 54F05-Gal4
S1a	59F02-Gal4 > UAS-CD4::CLIPf on 3rd	; ; UAS-CD4::CLIPf / 59F02-Gal4
S1a	50A02-Gal4 > UAS-mCD8::GFP	50A02-Gal4 / UAS-IVS-mCD8::GFP in attP2
S1a	54F05-Gal4 > UAS-mCD8::GFP	54F05-Gal4 / UAS-IVS-mCD8::GFP in attP2
S1a	59F02-Gal4 > UAS-mCD8::GFP	59F02-Gal4 / UAS-IVS-mCD8::GFP in attP2
S1b	Mz19-Gal4 > UAS-myr::4xCLIPf in attP40	; Mz19-Gal4 / UAS-myr::4xCLIPf in attP40 ;
S1b	50A02-Gal4 > UAS-myr::4xCLIPf in attP40	; UAS-myr::4xCLIPf in attP40 / + ; 50A02-Gal4 / +
S1b	54F05-Gal4 > UAS-myr::4xCLIPf in attP40	; UAS-myr::4xCLIPf in attP40 / + ; 54F05-Gal4 / +
S1b	59F02-Gal4 > UAS-myr::4xCLIPf in attP40	; UAS-myr::4xCLIPf in attP40 / + ; 59F02-Gal4 / +
S1b	Mz19-Gal4 > UAS-myr::4xCLIPf in VK00005	; Mz19-Gal4 / + ; UAS-myr::4xCLIPf in VK00005 / +
S1b	50A02-Gal4 > UAS-myr::4xCLIPf in VK00005	; ; UAS-myr::4xCLIPf in VK00005 / 50A02-Gal4
S1b	54F05-Gal4 > UAS-myr::4xCLIPf in VK00005	; ; UAS-myr::4xCLIPf in VK00005 / 54F05-Gal4
S1b	59F02-Gal4 > UAS-myr::4xCLIPf in VK00005	; ; UAS-myr::4xCLIPf in VK00005 / 59F02-Gal4
S1b	50A02-Gal4 > UAS-mCD8::GFP	; ; 50A02-Gal4 / UAS-IVS-mCD8::GFP in attP2

Figure	Genotype in figure	Full Genotype
S1b	54F05-Gal4 > UAS-mCD8::GFP	; ; 54F05-Gal4 / UAS-IVS-mCD8::GFP in attP2
S1b	59F02-Gal4 > UAS-mCD8::GFP	; ; 59F02-Gal4 / UAS-IVS-mCD8::GFP in attP2
S2a	Mz19-Gal4 > UAS-myr::4xSNAPf in attP40	; Mz19-Gal4 / UAS-myr::4xSNAPf in attP40 ;
S2a	54F05-Gal4 > UAS-myr::4xSNAPf in attP40	; UAS-myr::4xSNAPf in attP40 / + ; 54F05-Gal4 / +
S2a	59F02-Gal4 > UAS-myr::4xSNAPf in attP40	; UAS-myr::4xSNAPf in attP40 / + ; 59F02-Gal4 / +
S2a	54F05-Gal4 > UAS-mCD8::GFP	; ; 54F05-Gal4 / UAS-IVS-mCD8::GFP in attP2
S2a	59F02-Gal4 > UAS-mCD8::GFP	; ; 59F02-Gal4 / UAS-IVS-mCD8::GFP in attP2
S2b	Mz19-Gal4 > UAS-myr::4xSNAPf in attP2	; Mz19-Gal4 / + ; UAS-myr::4xSNAPf in attP2 / + ;
S2b	54F05-Gal4 > UAS-myr::4xSNAPf in attP2	; ; 54F05-Gal4 / UAS-myr::4xSNAPf in attP2
S2b	59F02-Gal4 > UAS-myr::4xSNAPf in attP2	; ; 59F02-Gal4 / UAS-myr::4xSNAPf in attP2
S2b	54F05-Gal4 > UAS-mCD8::GFP	; ; 54F05-Gal4 / UAS-IVS-mCD8::GFP in attP2
S2b	59F02-Gal4 > UAS-mCD8::GFP	; ; 59F02-Gal4 / UAS-IVS-mCD8::GFP in attP2
S2c	GH146-LexA > LexAop2-myr::4xCLIPf in VK00005	GH146-LexA / CyO ; LexAop2-myr::4xCLIPf in VK00005 / +
S2c	GH146-LexA > LexAop2-myr::4xSNAPf in VK00005	GH146-LexA / CyO ; LexAop2-myr::4xSNAPf in VK00005 / +
S3a	OR22a-Gal4 > UAS-mCD8::GFP	; OR22a-Gal4 / UAS-mCD8::GFP ;
S3a	OR22a-Gal4 > ; OR22a-Gal4 / UAS-mCD8::GFP ;	; OR22a-Gal4 / ; OR22a-Gal4 / UAS-mCD8::GFP in attP40 ;

Table S3: **Genotypes of flies used in each figure.** Transgenes marked with an * are not required nor have an effect on the experiment.

Figures

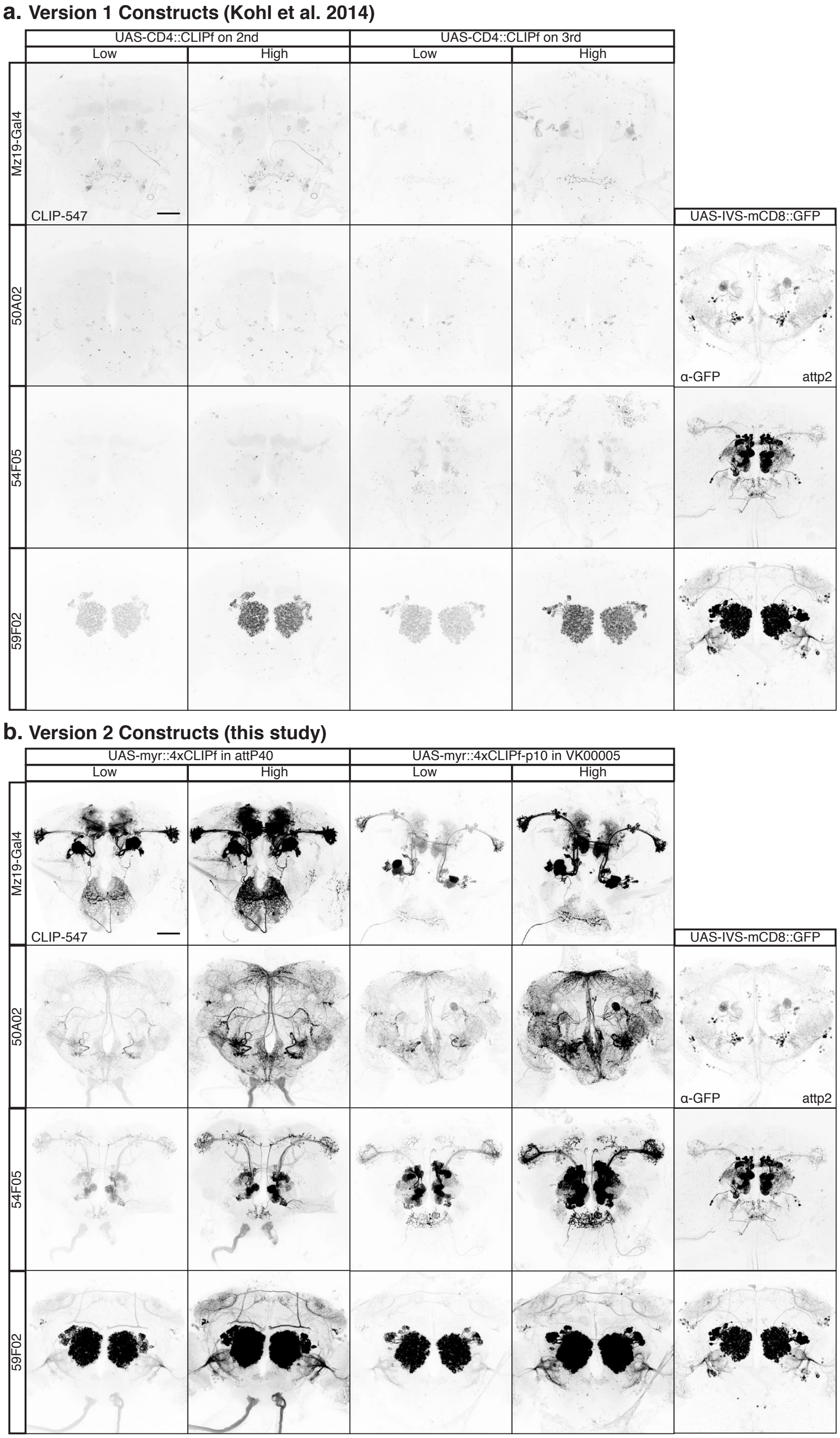

Figure S1: Figure legend on next page

Figure S1: **Labeling Gal4 drivers with old and new CLIPf reporters.** (**a**) Comparison of UAS-CD4::CLIPf, on 2nd and 3rd chromosomes from Kohl et al. (2014), labeling neurons in the Mz19-Gal4, GMR-50A02-Gal4, GMR-59F02-Gal4 and GMR-54F05-Gal4 expression patterns. The right most panels show the labeling of GMR-50A02-Gal4, GMR-59F02-Gal4 and GMR-54F05-Gal4 neurons using UAS-IVS-mCD8::GFP in attP2. (**b**) Comparison of UAS-myr::4xCLIPf, in attP40 and VK00005, labeling neurons in the Mz19-Gal4, GMR-50A02-Gal4, GMR-59F02-Gal4 and GMR-54F05-Gal4 expression patterns. The right most panels again show the labeling of GMR-50A02-Gal4, GMR-59F02-Gal4 and GMR-54F05-Gal4 neurons using UAS-IVS-mCD8::GFP in attP2. All images of chemical tagging reporters taking using the same confocal settings which achieved non-saturated images with the new transgenes. Right-most panels showing GFP staining are reproduced from http://flweb.janelia.org/cgi-bin/flew.cgi and were published in Jenett et al. (2012). “Low” and “High” refer to images acquired at two different laser power settings (low 2% and high 10%, see Methods). All scale bars are 50 µm.

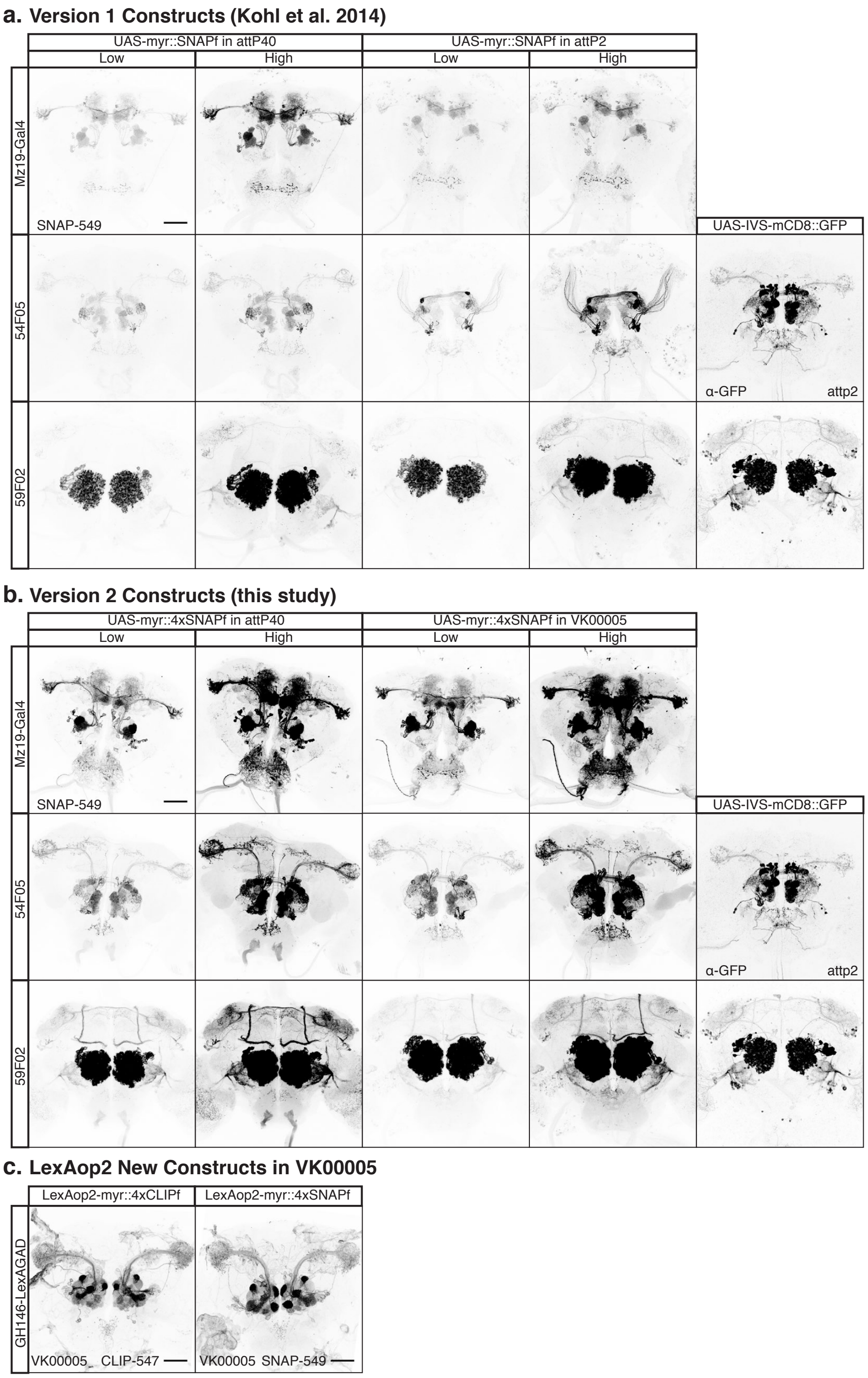

Figure S2: Figure legend on next page

Figure S2: **Labeling of Gal4 drivers with old and new SNAPf reporters.** (**a**) Comparison of version UAS-myr::SNAPf, in attP40 and attP2 from Kohl et al. (2014), labeling neurons in the Mz19-Gal4, GMR-59F02-Gal4 and GMR-54F05-Gal4 expression patterns. The right most panels show the labeling of GMR-59F02-Gal4 and GMR-54F05-Gal4 neurons using UAS-IVS-mCD8::GFP in attP2. (**b**) Comparison of UAS-myr::4xSNAPf, in attP40 and VK00005, labeling neurons in the Mz19-Gal4, GMR-59F02-Gal4 and GMR-54F05-Gal4 expression patterns. The right most panels again show the labeling of GMR-59F02-Gal4 and GMR-54F05-Gal4 neurons using UAS-IVS-mCD8::GFP in attP2. All images of chemical tagging reporters were aquired using the same confocal settings which achieved non-saturated images with the new transgenes. Right-most panels showing GFP staining are reproduced from http://flweb.janelia.org/cgi-bin/flew.cgi and were published in Jenett et al. (2012). "Low" and "High" refer to images acquired at two different laser power settings (low 2% and high 10%, see Methods). All scale bars are 50 µm.

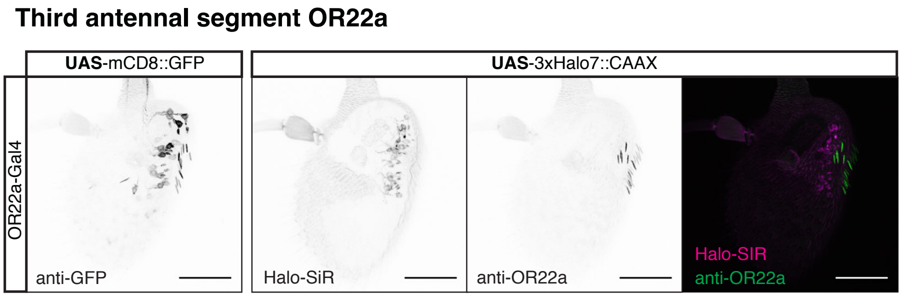

Figure S3: **Combining chemical labeling and antibody staining in the antennae.** Left panel shows staining of mCD8::GFP in Olfactory Receptor 22a expressing sensory neurons (OR22a). Next three panels show chemical labeling of cell membranes and antibody staining of the OR22a receptor. All panels partial projections of confocal stacks that exclude the cuticle. All inset images are the corresponding confocal full projections. All scale bars are 50 µm.

HeatShock-Bxb1-SV40

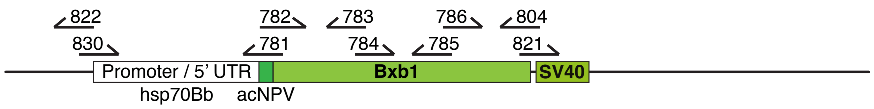

Primer	Sequence	Template
821	aatgagctaaggtctggtgttctagaggatctttgtgaaggaac	pJFRC161
822	gagcttaagactggccgtcgacctgcaggcatgcaagc	
830	cgacggccagtcttaagctcctagaatcccaaaacaaactggttattg	pCASPER-hs
781	ggctcgcattttgttatataatttgtaatttattcagagttctcttcttgtattcaat	
782	aattacaaattatataacaaaatgcgagccctggtcgtcatccgtttgtcccgtgtcac	Bxb1 gene synthesis block 1
783	agacgccactctccatccaca	
784	tgtggatggagagtggcgtct	Bxb1 gene synthesis block 2
785	gcttcgggaatcccatggaa	
786	ttccatgggattcccgaagc	Bxb1 gene synthesis block 3
804	acaccagaccttagctcattccggtgtgcaaacgctcgaccacgctgcccag	

Figure S4: **HeatShock-Bxb1-SV40.**

UAS-myr::>HA-BxbSTOP>SNAPf>CLIPf-p10

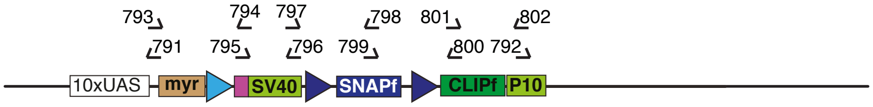

Primer	Sequence	Template
791	cttcttggcagatttcagtagttgcagttg	pJFRC81
792	caaatcaattgttttataatattcgtacgattctttg	
793	caactgcaactactgaaatctgcc	acNPV-myr::attP-HA gene synthesis block
794	cttgatatcgaattcttaagcgtaatctgg	
795	ccagattacgcttaagaattcgatatcaag	QUAS-mtdTomato
796	tgctttatttgtgaaatttgtgatgctattg	
797	caatagcatcacaaatttcacaaataaagca	SV40 UTR-attb-SNAPf gene synthesis block
798	cagcgaggtggctgtagctgatgacctctcc	
799	ggagaggtcatcagctacagccacctcgctg	SNAPf-attb-CLIPf gene synthesis block
800	ctggtggaagtaagcgttcaaccaag	
801	cttggttgaacgcttacttccaccag	CLIPf-p10 gene synthesis block
802	caaagaatcgtacgaatattataaaacaat	

UAS-myr::>HA-BxbSTOP>SNAPf-p10>CLIPf-p10

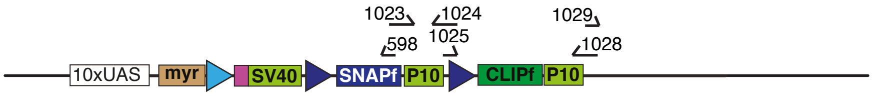

Primer	Sequence	Template
598	ttaacccagcccaggcttgc	UAS-myr::>HA-BxbSTOP> SNAPf>CLIPf-p10
1029	ttgaagacgaaagggcctc	
1023	caagcctgggctgggttaaatgaatcgtttttaaaataacaaatcaattg	UAS-myr::>HA-BxbSTOP> SNAPf>CLIPf-p10
1024	gacaagccgaacctaccttttgttaactcgaatcgctatccaagc	
1025	aaaaggtaggttcggcttgtc	UAS-myr::>HA-BxbSTOP> SNAPf>CLIPf-p10
1028	gaggccctttcgtcttcaagttaactcgaatcgctatccaag	

UAS-myr::>HA-BxbSTOP>SNAPf>CLIPf>Halo2-p10

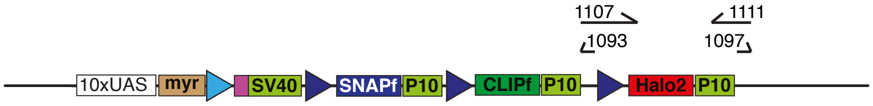

Primer	Sequence	Template
1093	cgaggccctttcgtcttcaag	UAS-myr::>HA-BxbSTOP> SNAPf-p10>CLIPf-p10
1097	ggttaatgtcatgataataatggtttcttag	
1107	gaagacgaaagggcctcgcggcttgtcgacgacggcggtctccgtcgtcag gatcatcttatgggttccgaaatcggtacag	pUAST-myr::Halo2
1111	gaaaccattattatcatgacattaaccgttaactcgaatcgctatccaag	

Figure S5: **UAS-myr::>HA-BxbSTOP>SNAPf>CLIPf>Halo2**

UAS-myr::4xCLIPf

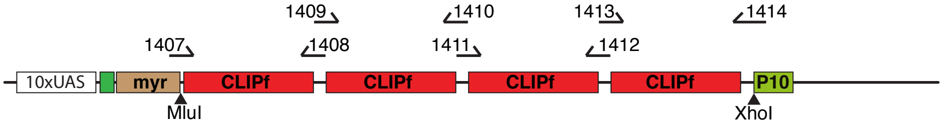

Primer	Sequence	Template
-	Double digest with MluI and XhoI enzymes to generate sticky ends.	UAS-myr::>HA-BxbSTOP> SNAPf-p10>CLIPf-p10>Halo2-p10
1407	gacatcatcagaccacgcgtgatggataaggattgcgagatgaag	UAS-myr::>HA-BxbSTOP> SNAPf-p10>CLIPf-p10>Halo2-p10
1408	tgtactaccgcttacgcttcccaagccgggctttcc	
1409	agcgtaagcggtagtacaatggataaggattgcgagatgaag	UAS-myr::>HA-BxbSTOP> SNAPf-p10>CLIPf-p10>Halo2-p10
1410	ggtactgctaccgcttactcccaagccgggctttcc	
1411	ggagtaagcggtagcagtaccatggataaggattgcgagatgaag	UAS-myr::>HA-BxbSTOP> SNAPf-p10>CLIPf-p10>Halo2-p10
1412	cgtgctactactaccaaccgatcccaagccgggctttcc	
1413	tcggttggtagtagtagcacgatggataaggattgcgagatgaag	UAS-myr::>HA-BxbSTOP> SNAPf-p10>CLIPf-p10>Halo2-p10
1414	gttattttaaaaacgattcattctagattaactagtctcgagtcactatccca agccgggctttcc	

Figure S6: **UAS-myr::4xCLIPf**

UAS-myr::4xSNAPf

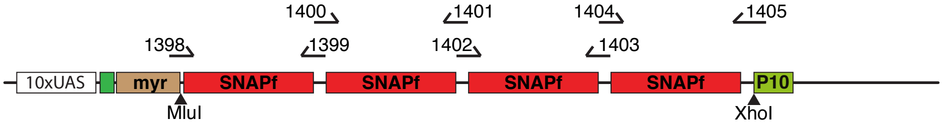

Primer	Sequence	Template
	Double digest with MluI and XhoI enzymes to generate sticky ends.	UAS-myr::>HA-BxbSTOP> SNAPf-p10>CLIPf-p10>Halo2-p10
1398	gacatcatcagaccacgcgtgatggacaaagactgcgaaatgaag	UAS-myr::>HA-BxbSTOP> SNAPf-p10>CLIPf-p10>Halo2-p10
1399	tgtactaccgcttacgctacccagcccaggcttgc	
1400	agcgtaagcggtagtacaatggacaaagactgcgaaatgaag	UAS-myr::>HA-BxbSTOP> SNAPf-p10>CLIPf-p10>Halo2-p10
1401	ggtactgctaccgcttacacccagcccaggcttgc	
1402	ggtgtaagcggtagcagtaccatggacaaagactgcgaaatgaag	UAS-myr::>HA-BxbSTOP> SNAPf-p10>CLIPf-p10>Halo2-p10
1403	cgtgctactactaccaaccgaacccagcccaggcttgc	
1404	tcggttggtagtagtagcacgatggacaaagactgcgaaatgaag	UAS-myr::>HA-BxbSTOP> SNAPf-p10>CLIPf-p10>Halo2-p10
1405	gttattttaaaaacgattcattctagattaactagtctcgagtcactaaccc agcccaggcttgc	

Figure S7: **UAS-myr::4xSNAPf**

LexAop2-myr::4xCLIPf

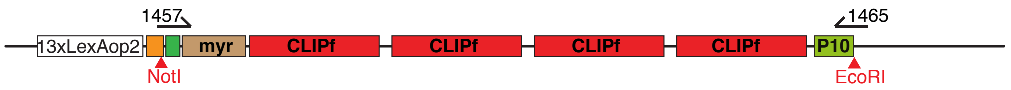

Primer	Sequence	Template
-	Double digest with NotI and EcoRI enzymes to generate sticky ends.	pJFRC19
1457	ccctaattcttatcctttacttcaggcaattacaaattatataacaaaatggg caacaaatgctgc	UAS-myr::4xCLIPf
1465	gaggccctttcgtcttcaaggttaactcgaatcgctatccaagccag	

Figure S8: **LexAop2-myr::4xCLIPf.** Red restriction enzymes indicate that the site is destroyed during the assembly reaction.

LexAop2-myr::4xSNAPf

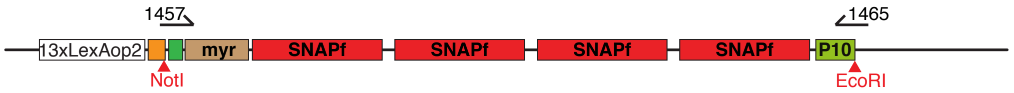

Primer	Sequence	Template
-	Double digest with NotI and EcoRI enzymes to generate sticky ends.	pJFRC19
1457	ccctaattcttatcctttacttcaggcaattacaaattatataacaaaatggg caacaaatgctgc	UAS-myr::4xSNAPf
1465	gaggccctttcgtcttcaaggttaactcgaatcgctatccaagccag	

Figure S9: **LexAop2-myr::4xSNAPf**

UAS-myr::>HA-BxbSTOP>4xSNAPf

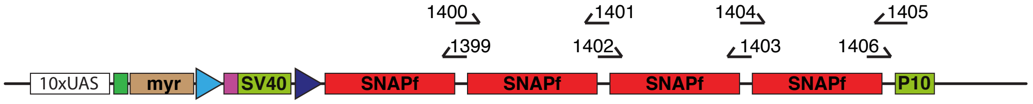

Primer	Sequence	Template
1399	tgtactaccgcttacgctacccagcccaggcttgc	UAS-myr::>HA-BxbSTOP>SNAPf -p10>CLIPf-p10>Halo2-p10
1406	tagtgactcgagactagttaatctagaatgaatcg	
1400	agcgtaagcggtagtacaatggacaaagactgcgaaatgaag	UAS-myr::>HA-BxbSTOP>SNAPf -p10>CLIPf-p10>Halo2-p10
1401	ggtactgctaccgcttacacccagcccaggcttgc	
1402	ggtgtaagcggtagcagtaccatggacaaagactgcgaaatgaag	UAS-myr::>HA-BxbSTOP>SNAPf-p10>CLIPf-p10>Halo2-p10
1403	cgtgctactactaccaaccgaacccagcccaggcttgc	
1404	tcggttggtagtagtagcacgatggacaaagactgcgaaatgaag	UAS-myr::>HA-BxbSTOP>SNAPf-p10>CLIPf-p10>Halo2-p10
1405	gttattttaaaaacgattcattctagattaactagtctcgagtcactaaccca gcccaggcttgc	

Figure S10: **UAS-myr::>HA-BxbSTOP>4xSNAPf**

LexAop2-myr::>HA-BxbSTOP>4xSNAPf

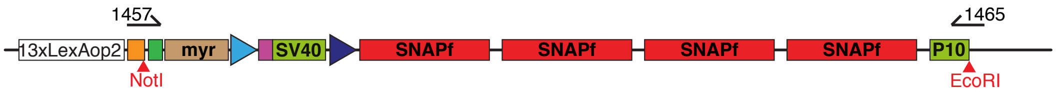

Primer	Sequence	Template
-	Double digest with NotI and EcoRI enzymes to generate sticky ends.	pJFRC19
1457	ccctaattcttatcctttacttcaggcaattacaaattatataacaaaatggg caacaaatgctgc	UAS-myr::>BxbSTOP>4xSNAPf
1465	gaggccctttcgtcttcaaggttaactcgaatcgctatccaagccag	

Figure S11: **LexAop2-myr::>HA-BxbSTOP>4xSNAPf**

HeatShock-Bxb1

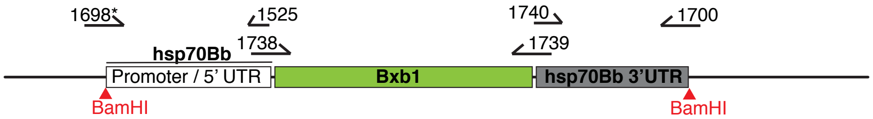

Primer	Sequence	Template
-	Digest with BamHI enzyme to generate sticky ends.	pUAST-TLN-cherry (Nicolaï et al., 2010)
1698	aagcttctgcatctctccgctagaatcccaaaacaaactggttattgtgg	heatshock-Bxb-SV40 3' UTR
1525	tattcagagttctcttcttgtattcaataattac	
1738	gtaattattgaatacaagaagagaactctgaatacaaaatgcgagccctggtcgtc	heatshock-Bxb-SV40 3' UTR
1739	gaactgaataattttctatttggctttagtcttagctcattccggtgtgcaaac	
1740	gactaaagccaaatagaaaattattcagttcc	Fly genomic DNA
1700	catacatactaaggccttctagtggatctaaacgagtttttaagcaaac	

Figure S12: **HeatShock-Bxb1**. Red restriction enzymes indicate that the site is destroyed during the assembly reaction. Part of the sequence for primer 1698* was not found on the cloned construct; the difference being upstream of the functional sequences does not affect its activity.

UAS->FlpSTOP>Bxb1

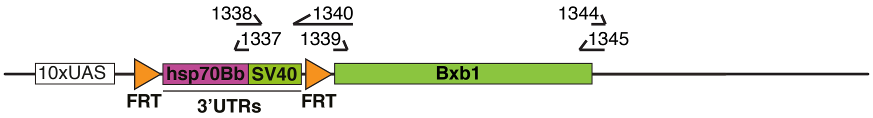

UAS->Bxb1

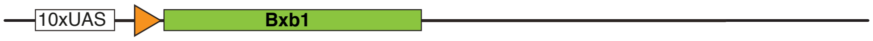

Primer	Sequence	Template
1344	gtacaaagtggtgggtaccggatc	pJFRC-MUH-FRT-hsp70Bb-FRT (unpublished)
1337	gccggccgcaactagaactag	
1338	ctagttctagttgcggccggctaagaattcgatatcaagcttatcgataccg	UAS-myr::>HA-BxbSTOP> SNAPf-p10>CLIPf-p10>Halo2-p10
1340	gacgaccagggctcgcattttggaagttcctatactttctagagaataggaa cttcgatccagacatgataagatacattgatgagtttg	
1339	atgcgagccctggtcgtc	heatshock-Bxb-SV40 3' UTR
1345	cggtacccaccactttgtacttagctcattccggtgtgcaaac	

Figure S13: **UAS->FlpSTOP>Bxb1**. UAS->Bxb1 was obtained by activating UAS-FLP on the germ line of males using nanos-Gal4. Flp recombination induces removal of the stop cassette in the germ line and allowed the establishment of a stock.

pJET p1.2-1xHalo7::CAAX

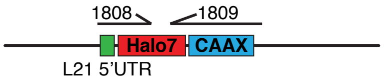

Cloning steps
Syn21-Halo7::CAAX PCR product (see table below) was blunt-end ligated into vector pJET p1.2 (ThermoFisher Scientific).

Primer	Sequence	Template
1808	ttcaggcggccgcaactcctaaaaaaccgccaccatggcagaaatcggtactggc	pHTN-JF920304; Promega Inc.
1809	ccctctagattacataattacacactttgtctttgacttctttttcttctttttac catctttgctcatctcgagactagtagatctgccggaaatttcgagcgtcgaca	

UAS-1xHalo7::CAAX

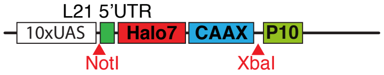

Cloning steps
- Following sequence verification, pJET p1.2-1xHalo7::CAAX was digested with Not1 and Xba1.
- The Halo7::CAAX fragment was ligated into the Drosophila transformation vector, pJFRC81 using Not1 and Xba1 sites.

Figure S14: **UAS-1xHalo7::CAAX**.

pJET p1.2-1xHalo7

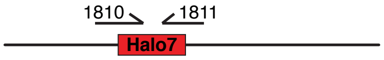

Cloning steps
Halo7 PCR product (see table below) was blunt-end ligated into vector pJET p1.2 (ThermoFisher Scientific).

Primer	Sequence	Template
1810	gggaccggttctagaggatccatggcagaaatcggtactggc	pHTN-JF920304; Promega Inc.
1811	ccctctagattactcgagactagtagatctgccggaaatttcgagcgtcgaca gccagcg	

pJET p1.2-2xHalo7

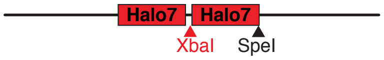

Cloning steps
- Following sequence verification, pJET p1.2-1xHalo7 was digested with with XbaI and SpeI.
- The Halo fragment was re-inserted into pJET p1.2-1xHalo7 digested with SpeI. During this step the XbaI site (red) combines with the SpeI site and gets destroyed while one SpeI site gets retained.

UAS-3xHalo7::CAAX

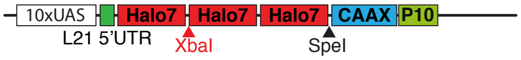

Cloning steps
- pJET p1.2-2xHalo7 was digested with Xba1 and Spe1 and the 2xHalo fragment was purified.
- UAS-1xHalo7::CAAX was treated with SpeI and the 2xHalo fragment ligated. During the cloning the XbaI site gets destroyed and the SpeI site is retained.

Figure S15: **UAS-3xHalo7::CAAX.**

pJET p1.2-4xHalo7

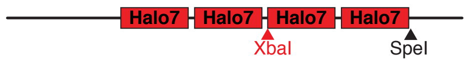

Cloning steps
- Following sequence verification, pJET p1.2-2xHalo7 was digested with with XbaI and SpeI.
- The 2xHalo fragment was re-inserted into pJET p1.2-2xHalo7 digested with SpeI. During this step the XbaI site (red) combines with the SpeI site and gets destroyed while one SpeI site gets retained.

UAS-7xHalo7::CAAX

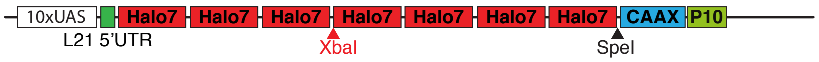

Cloning steps
- pJET p1.2-4xHalo7 was digested with Xba1 and Spe1 and the 4xHalo fragment was purified.
- UAS-3xHalo7::CAAX was treated with SpeI and the 4xHalo fragment ligated. During the cloning the XbaI site gets destroyed and the SpeI site is retained.

Figure S16: **UAS-7xHalo7::CAAX.**

UAS-Synaptotagmin::1xHalo7

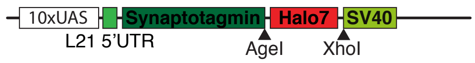

Cloning steps
- A previously made Drosophila transformation vector plasmid bearing the Synaptotagmin coding region, pJFRC81-Syt::TMP (unpublished), was digested with Age1 and Xho1.
- The purified pJFRC81-Syt was ligated with a Halo7 fragment from pJET 1.2-Halo7 digested with Age1 and Xho1 to create pJFRC81-Syt::Halo7.

Figure S17: **UAS-Synaptotagmin::1xHalo7**

UAS-Synaptotagmin::3xHalo7

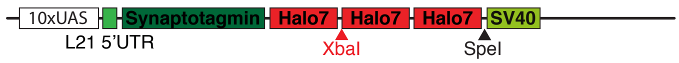

Cloning steps
- pJET p1.2-2xHalo7 was digested with Xba1 and Spe1 and the 2xHalo fragment was purified.
- UAS-Synaptotagmin::Halo was treated with SpeI and the 2xHalo fragment ligated. During the cloning the XbaI site gets destroyed and the SpeI site is retained.

Figure S18: **UAS-Synaptotagmin::3xHalo7**

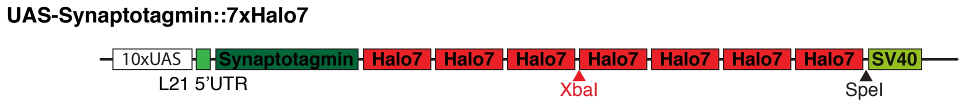

Cloning steps
- pJET p1.2-4xHalo7 was digested with Xba1 and Spe1 and the 4xHalo fragment was purified.
- UAS-Synaptotagmin::3xHalo was treated with SpeI and the 4xHalo fragment ligated. During the cloning the XbaI site gets destroyed and the SpeI site is retained.

Figure S19: **UAS-Synaptotagmin::7xHalo7**

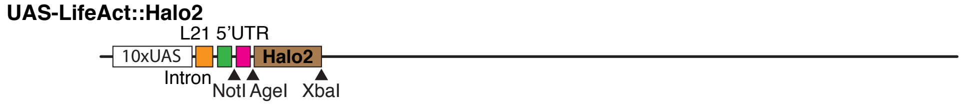

Cloning steps
- pJFRC81-myr::Halo2 Kohl et al. (2014) was digested with Xba1 and Age1 and purified.
- Halo2 fragment was ligated in a previously prepared pJFRC81-LifeAct digested with Xba1 and Age1.

Figure S20: **UAS-LA-Halo2**
