# Supplementary material for: Second-Generation Drosophila Chemical Tags: Sensitivity, Versatility, and Speed
Source: Genetics. 2017 Feb 14;205(4):1399–408. doi: 10.1534/genetics.116.199281 (PMC5378102; doi:10.1534/genetics.116.199281)
Supplement: Supplementary file 2 [file 1399FileS2.zip › R-Markdown.html]

AnalyseROIs


# AnalyseROIs

#### *Gregory Jefferis, Ben Sutcliffe, Sebastian Cachero*

#### *29 January 2016*

# Preface

This is an R Markdown document. Markdown is a simple formatting syntax for authoring HTML, PDF, and MS Word documents. For more details on using R Markdown see http://rmarkdown.rstudio.com.

When you click the **Knit** button a document will be generated that includes both content as well as the output of any embedded R code chunks within the document.

# Setup

```
library(nat)
```

```
## Loading required package: rgl
```

```
## 
## Attaching package: 'nat'
```

```
## The following objects are masked from 'package:base':
## 
##     intersect, setdiff, union
```

```
source("rfuns.R")
library(knitr)
```

# ROIs

We want to look at the ROIs for each image. Let’s first find the images

```
# Find the brains with ROIs
brains=unique(brain_from_path(dir("amira")))
lows=image_for_brain(brains, 'high')
braindf=data.frame(brain=brains, low=lows, 
              driver=extract_driver(lows),
              reporter=extract_reporter(lows),
              insertion=extract_insertion(lows))
write.csv(braindf, file='braindf.csv')
kable(braindf)
```

|  | brain | low | driver | reporter | insertion |
| --- | --- | --- | --- | --- | --- |
| BADF02 | BADF02 | /Volumes/JData/JPeople/Ben/Confocal/chemtag/BADF/nrrds/BADF02-Mz19-GJ851-UAS-CD4-CLIPf\_02.nrrd | MZ19 | CD4-CLIPf | GJ851 |
| BADF03 | BADF03 | /Volumes/JData/JPeople/Ben/Confocal/chemtag/BADF/nrrds/BADF03-Mz19-GJ851-UAS-CD4-CLIPf\_02.nrrd | MZ19 | CD4-CLIPf | GJ851 |
| BADF05 | BADF05 | /Volumes/JData/JPeople/Ben/Confocal/chemtag/BADF/nrrds/BADF05-Mz19-GJ851-UAS-CD4-CLIPf\_02.nrrd | MZ19 | CD4-CLIPf | GJ851 |
| BADF15 | BADF15 | /Volumes/JData/JPeople/Ben/Confocal/chemtag/BADF/nrrds/BADF15-Mz19-GJ851-UAS-CD4-CLIPf\_02.nrrd | MZ19 | CD4-CLIPf | GJ851 |
| BADH01 | BADH01 | /Volumes/JData/JPeople/Ben/Confocal/chemtag/BADH/nrrds/BADH01-mz19-15137-1-2M-UAS-myr-4xCLIPf-VK5\_02.nrrd | MZ19 | myr-4xCLIPf | VK5 |
| BADH11 | BADH11 | /Volumes/JData/JPeople/Ben/Confocal/chemtag/BADH/nrrds/BADH11-mz19-15137-1-2M-UAS-myr-4xCLIPf-VK5\_02.nrrd | MZ19 | myr-4xCLIPf | VK5 |
| BADH13 | BADH13 | /Volumes/JData/JPeople/Ben/Confocal/chemtag/BADH/nrrds/BADH13-mz19-15137-1-2M-UAS-myr-4xCLIPf-VK5\_02.nrrd | MZ19 | myr-4xCLIPf | VK5 |
| BADH15 | BADH15 | /Volumes/JData/JPeople/Ben/Confocal/chemtag/BADH/nrrds/BADH15-mz19-15137-1-2M-UAS-myr-4xCLIPf-VK5\_02.nrrd | MZ19 | myr-4xCLIPf | VK5 |
| BADS04 | BADS04 | /Volumes/JData5/JPeople/Ben/Confocal/chemtag/BADS/nrrds/BADS04\_MZ19-15138-1-1M\_UAS-myr-4xCLIPf-attP40-CLIP-547\_02.nrrd | MZ19 | myr-4xCLIPf | P40 |
| BADS05 | BADS05 | /Volumes/JData5/JPeople/Ben/Confocal/chemtag/BADS/nrrds/BADS05\_MZ19-15138-1-1M\_UAS-myr-4xCLIPf-attP40-CLIP-547\_02.nrrd | MZ19 | myr-4xCLIPf | P40 |
| BADS06 | BADS06 | /Volumes/JData5/JPeople/Ben/Confocal/chemtag/BADS/nrrds/BADS06\_MZ19-15138-1-1M\_UAS-myr-4xCLIPf-attP40-CLIP-547\_02.nrrd | MZ19 | myr-4xCLIPf | P40 |
| BADS07 | BADS07 | /Volumes/JData5/JPeople/Ben/Confocal/chemtag/BADS/nrrds/BADS07\_MZ19-15138-1-1M\_UAS-myr-4xCLIPf-attP40-CLIP-547\_02.nrrd | MZ19 | myr-4xCLIPf | P40 |
| BADS10 | BADS10 | /Volumes/JData5/JPeople/Ben/Confocal/chemtag/BADS/nrrds/BADS10\_MZ19-GJ853-UAS-CD4-CLIPf-CLIP-547\_02.nrrd | MZ19 | CD4-CLIPf | GJ853 |
| BADS11 | BADS11 | /Volumes/JData5/JPeople/Ben/Confocal/chemtag/BADS/nrrds/BADS11\_MZ19-GJ853-UAS-CD4-CLIPf-CLIP-547\_02.nrrd | MZ19 | CD4-CLIPf | GJ853 |
| BADS13 | BADS13 | /Volumes/JData5/JPeople/Ben/Confocal/chemtag/BADS/nrrds/BADS13\_MZ19-GJ853-UAS-CD4-CLIPf-CLIP-547\_02.nrrd | MZ19 | CD4-CLIPf | GJ853 |
| BAER01 | BAER01 | /Volumes/JData5/JPeople/Ben/Confocal/chemtag/BAER/nrrds/BAER01\_MZ19-GJ838-UAS-myr-SNAPf-attP2-SNAP-549\_02.nrrd | MZ19 | myr-SNAPf | P2 |
| BAER02 | BAER02 | /Volumes/JData5/JPeople/Ben/Confocal/chemtag/BAER/nrrds/BAER02\_MZ19-GJ838-UAS-myr-SNAPf-attP2-SNAP-549\_02.nrrd | MZ19 | myr-SNAPf | P2 |
| BAER03 | BAER03 | /Volumes/JData5/JPeople/Ben/Confocal/chemtag/BAER/nrrds/BAER03\_MZ19-GJ838-UAS-myr-SNAPf-attP2-SNAP-549\_02.nrrd | MZ19 | myr-SNAPf | P2 |
| BAER04 | BAER04 | /Volumes/JData5/JPeople/Ben/Confocal/chemtag/BAER/nrrds/BAER04\_MZ19-GJ838-UAS-myr-SNAPf-attP2-SNAP-549\_02.nrrd | MZ19 | myr-SNAPf | P2 |
| BAER09 | BAER09 | /Volumes/JData5/JPeople/Ben/Confocal/chemtag/BAER/nrrds/BAER09\_MZ19-GJ865-UAS-myr-SNAPf-attP40-SNAP-549\_02.nrrd | MZ19 | myr-SNAPf | P40 |
| BAER10 | BAER10 | /Volumes/JData5/JPeople/Ben/Confocal/chemtag/BAER/nrrds/BAER10\_MZ19-GJ865-UAS-myr-SNAPf-attP40-SNAP-549\_02.nrrd | MZ19 | myr-SNAPf | P40 |
| BAER11 | BAER11 | /Volumes/JData5/JPeople/Ben/Confocal/chemtag/BAER/nrrds/BAER11\_MZ19-GJ865-UAS-myr-SNAPf-attP40-SNAP-549\_02.nrrd | MZ19 | myr-SNAPf | P40 |
| BAER12 | BAER12 | /Volumes/JData5/JPeople/Ben/Confocal/chemtag/BAER/nrrds/BAER12\_MZ19-GJ865-UAS-myr-SNAPf-attP40-SNAP-549\_02.nrrd | MZ19 | myr-SNAPf | P40 |
| BAER13 | BAER13 | /Volumes/JData5/JPeople/Ben/Confocal/chemtag/BAER/nrrds/BAER13\_MZ19-GJ865-UAS-myr-SNAPf-attP40-SNAP-549\_02.nrrd | MZ19 | myr-SNAPf | P40 |
| BAER14 | BAER14 | /Volumes/JData5/JPeople/Ben/Confocal/chemtag/BAER/nrrds/BAER14\_MZ19-15138-2-1M-T07-UAS-myr-4xSNAPf-attP40-SNAP-549\_02.nrrd | MZ19 | myr-4xSNAPf | P40 |
| BAER15 | BAER15 | /Volumes/JData5/JPeople/Ben/Confocal/chemtag/BAER/nrrds/BAER15\_MZ19-15138-2-1M-T07-UAS-myr-4xSNAPf-attP40-SNAP-549\_02.nrrd | MZ19 | myr-4xSNAPf | P40 |
| BAER16 | BAER16 | /Volumes/JData5/JPeople/Ben/Confocal/chemtag/BAER/nrrds/BAER16\_MZ19-15138-2-1M-T07-UAS-myr-4xSNAPf-attP40-SNAP-549\_02.nrrd | MZ19 | myr-4xSNAPf | P40 |
| BAER17 | BAER17 | /Volumes/JData5/JPeople/Ben/Confocal/chemtag/BAER/nrrds/BAER17\_MZ19-15138-2-1M-T07-UAS-myr-4xSNAPf-attP40-SNAP-549\_02.nrrd | MZ19 | myr-4xSNAPf | P40 |
| BAER19 | BAER19 | /Volumes/JData5/JPeople/Ben/Confocal/chemtag/BAER/nrrds/BAER19\_MZ19-15137-2-2M-T02-UAS-myr-4xSNAPf-VK5-SNAP-549\_02.nrrd | MZ19 | myr-4xSNAPf | VK5 |
| BAER20 | BAER20 | /Volumes/JData5/JPeople/Ben/Confocal/chemtag/BAER/nrrds/BAER20\_MZ19-15137-2-2M-T02-UAS-myr-4xSNAPf-VK5-SNAP-549\_02.nrrd | MZ19 | myr-4xSNAPf | VK5 |
| BAER21 | BAER21 | /Volumes/JData5/JPeople/Ben/Confocal/chemtag/BAER/nrrds/BAER21\_MZ19-15137-2-2M-T02-UAS-myr-4xSNAPf-VK5-SNAP-549\_02.nrrd | MZ19 | myr-4xSNAPf | VK5 |
| BAER22 | BAER22 | /Volumes/JData5/JPeople/Ben/Confocal/chemtag/BAER/nrrds/BAER22\_MZ19-15137-2-2M-T02-UAS-myr-4xSNAPf-VK5-SNAP-549\_02.nrrd | MZ19 | myr-4xSNAPf | VK5 |
| BAER23 | BAER23 | /Volumes/JData5/JPeople/Ben/Confocal/chemtag/BAER/nrrds/BAER23\_MZ19-15137-2-2M-T02-UAS-myr-4xSNAPf-VK5-SNAP-549\_02.nrrd | MZ19 | myr-4xSNAPf | VK5 |
| BAFF01 | BAFF01 | /Volumes/JData5/JPeople/Ben/Confocal/chemtag/BAFF/nrrds/BAFF01\_MZ19-UAS-Halo7-CAAX-P40-1\_02.nrrd | MZ19 | UAS-Halo7-CAAX | P40 |
| BAFF02 | BAFF02 | /Volumes/JData5/JPeople/Ben/Confocal/chemtag/BAFF/nrrds/BAFF02\_MZ19-UAS-Halo7-CAAX-P40-1\_02.nrrd | MZ19 | UAS-Halo7-CAAX | P40 |
| BAFF03 | BAFF03 | /Volumes/JData5/JPeople/Ben/Confocal/chemtag/BAFF/nrrds/BAFF03\_MZ19-UAS-Halo7-CAAX-P40-1\_02.nrrd | MZ19 | UAS-Halo7-CAAX | P40 |
| BAFF04 | BAFF04 | /Volumes/JData5/JPeople/Ben/Confocal/chemtag/BAFF/nrrds/BAFF04\_MZ19-UAS-Halo7-CAAX-P40-1\_02.nrrd | MZ19 | UAS-Halo7-CAAX | P40 |
| BAFF05 | BAFF05 | /Volumes/JData5/JPeople/Ben/Confocal/chemtag/BAFF/nrrds/BAFF05\_MZ19-UAS-Halo7-CAAX-P40-1\_02.nrrd | MZ19 | UAS-Halo7-CAAX | P40 |
| BAFF06 | BAFF06 | /Volumes/JData5/JPeople/Ben/Confocal/chemtag/BAFF/nrrds/BAFF06\_MZ19-UAS-Halo7-CAAX-P40-1\_02.nrrd | MZ19 | UAS-Halo7-CAAX | P40 |
| BAFF07 | BAFF07 | /Volumes/JData5/JPeople/Ben/Confocal/chemtag/BAFF/nrrds/BAFF07\_MZ19-UAS-Halo7-CAAX-P40-1\_02.nrrd | MZ19 | UAS-Halo7-CAAX | P40 |
| BAFF08 | BAFF08 | /Volumes/JData5/JPeople/Ben/Confocal/chemtag/BAFF/nrrds/BAFF08\_MZ19-UAS-td3-Halo7-CAAX-P40-2\_02.nrrd | MZ19 | UAS-td3-Halo7-CAAX | P40 |
| BAFF09 | BAFF09 | /Volumes/JData5/JPeople/Ben/Confocal/chemtag/BAFF/nrrds/BAFF09\_MZ19-UAS-td3-Halo7-CAAX-P40-2\_02.nrrd | MZ19 | UAS-td3-Halo7-CAAX | P40 |
| BAFF10 | BAFF10 | /Volumes/JData5/JPeople/Ben/Confocal/chemtag/BAFF/nrrds/BAFF10\_MZ19-UAS-td3-Halo7-CAAX-P40-2\_02.nrrd | MZ19 | UAS-td3-Halo7-CAAX | P40 |
| BAFF11 | BAFF11 | /Volumes/JData5/JPeople/Ben/Confocal/chemtag/BAFF/nrrds/BAFF11\_MZ19-UAS-td3-Halo7-CAAX-P40-2\_02.nrrd | MZ19 | UAS-td3-Halo7-CAAX | P40 |
| BAFF12 | BAFF12 | /Volumes/JData5/JPeople/Ben/Confocal/chemtag/BAFF/nrrds/BAFF12\_MZ19-UAS-td3-Halo7-CAAX-P40-2\_02.nrrd | MZ19 | UAS-td3-Halo7-CAAX | P40 |
| BAFF13 | BAFF13 | /Volumes/JData5/JPeople/Ben/Confocal/chemtag/BAFF/nrrds/BAFF13\_MZ19-UAS-td3-Halo7-CAAX-P40-2\_02.nrrd | MZ19 | UAS-td3-Halo7-CAAX | P40 |
| BAFF14 | BAFF14 | /Volumes/JData5/JPeople/Ben/Confocal/chemtag/BAFF/nrrds/BAFF14\_MZ19-UAS-td3-Halo7-CAAX-P40-2\_02.nrrd | MZ19 | UAS-td3-Halo7-CAAX | P40 |
| BAFF15 | BAFF15 | /Volumes/JData5/JPeople/Ben/Confocal/chemtag/BAFF/nrrds/BAFF15\_MZ19-UAS-td3-Halo7-CAAX-P40-2\_02.nrrd | MZ19 | UAS-td3-Halo7-CAAX | P40 |
| BAFF16 | BAFF16 | /Volumes/JData5/JPeople/Ben/Confocal/chemtag/BAFF/nrrds/BAFF16\_MZ19-UAS-td7-Halo7-CAAX-P40-1\_02.nrrd | MZ19 | UAS-td7-Halo7-CAAX | P40 |
| BAFF17 | BAFF17 | /Volumes/JData5/JPeople/Ben/Confocal/chemtag/BAFF/nrrds/BAFF17\_MZ19-UAS-td7-Halo7-CAAX-P40-1\_02.nrrd | MZ19 | UAS-td7-Halo7-CAAX | P40 |
| BAFF18 | BAFF18 | /Volumes/JData5/JPeople/Ben/Confocal/chemtag/BAFF/nrrds/BAFF18\_MZ19-UAS-td7-Halo7-CAAX-P40-1\_02.nrrd | MZ19 | UAS-td7-Halo7-CAAX | P40 |
| BAFF19 | BAFF19 | /Volumes/JData5/JPeople/Ben/Confocal/chemtag/BAFF/nrrds/BAFF19\_MZ19-UAS-td7-Halo7-CAAX-P40-1\_02.nrrd | MZ19 | UAS-td7-Halo7-CAAX | P40 |
| BAFF20 | BAFF20 | /Volumes/JData5/JPeople/Ben/Confocal/chemtag/BAFF/nrrds/BAFF20\_MZ19-UAS-td7-Halo7-CAAX-P40-1\_02.nrrd | MZ19 | UAS-td7-Halo7-CAAX | P40 |
| BAFF21 | BAFF21 | /Volumes/JData5/JPeople/Ben/Confocal/chemtag/BAFF/nrrds/BAFF21\_MZ19-UAS-td7-Halo7-CAAX-P40-1\_02.nrrd | MZ19 | UAS-td7-Halo7-CAAX | P40 |
| BAFF22 | BAFF22 | /Volumes/JData5/JPeople/Ben/Confocal/chemtag/BAFF/nrrds/BAFF22\_MZ19-UAS-td7-Halo7-CAAX-P40-1\_02.nrrd | MZ19 | UAS-td7-Halo7-CAAX | P40 |
| BAFF23 | BAFF23 | /Volumes/JData5/JPeople/Ben/Confocal/chemtag/BAFF/nrrds/BAFF23\_MZ19-UAS-td7-Halo7-CAAX-P40-1\_02.nrrd | MZ19 | UAS-td7-Halo7-CAAX | P40 |
| BAFF24 | BAFF24 | /Volumes/JData5/JPeople/Ben/Confocal/chemtag/BAFF/nrrds/BAFF24\_MZ19-UAS-myr-Halo2-P40\_02.nrrd | MZ19 | myr-Halo2 | P40 |
| BAFF25 | BAFF25 | /Volumes/JData5/JPeople/Ben/Confocal/chemtag/BAFF/nrrds/BAFF25\_MZ19-UAS-myr-Halo2-P40\_02.nrrd | MZ19 | myr-Halo2 | P40 |
| BAFF26 | BAFF26 | /Volumes/JData5/JPeople/Ben/Confocal/chemtag/BAFF/nrrds/BAFF26\_MZ19-UAS-myr-Halo2-P40\_02.nrrd | MZ19 | myr-Halo2 | P40 |
| BAFF27 | BAFF27 | /Volumes/JData5/JPeople/Ben/Confocal/chemtag/BAFF/nrrds/BAFF27\_MZ19-UAS-myr-Halo2-P40\_02.nrrd | MZ19 | myr-Halo2 | P40 |
| BAFF28 | BAFF28 | /Volumes/JData5/JPeople/Ben/Confocal/chemtag/BAFF/nrrds/BAFF28\_MZ19-UAS-myr-Halo2-P40\_02.nrrd | MZ19 | myr-Halo2 | P40 |
| BAFF29 | BAFF29 | /Volumes/JData5/JPeople/Ben/Confocal/chemtag/BAFF/nrrds/BAFF29\_MZ19-UAS-myr-Halo2-P40\_02.nrrd | MZ19 | myr-Halo2 | P40 |
| BAFF30 | BAFF30 | /Volumes/JData5/JPeople/Ben/Confocal/chemtag/BAFF/nrrds/BAFF30\_MZ19-UAS-myr-Halo2-P40\_02.nrrd | MZ19 | myr-Halo2 | P40 |

```
# Define a function to calculate the values that we want 
roi_values_brain <- function(brain, FUN=mean){
  print(paste("working on brain: ", brain, " filename:" ,image_for_brain(brain, 'high'),sep=""))
  orig=read.im3d(image_for_brain(brain, 'high'))
  labels=read.im3d(image_for_brain(brain, 'labels'))
  mats=materials(labels)
  our_mats=subset(mats, !name%in%c("Exterior", "Inside"))
  foreground=subset(our_mats, !grepl("bg-", name, fixed=T))
  background_names=paste0('bg-', foreground$name)
  if(!all(background_names%in%our_mats$name))
    stop("mismatch between foreground and background names!")
  
  foreground$foreground=NA_real_
  foreground$background=NA_real_
  for (mat in foreground$name){
    foreground[match(mat, foreground$name),'foreground']=FUN(mask(orig, labels, levels = mat, rval = 'values'))
    foreground[match(mat, foreground$name),'background']=FUN(mask(orig, labels, levels = paste0('bg-', mat), rval = 'values'))
  }
  foreground$brain=brain
  foreground
}
```

Do the main computation

```
brain_results=lapply(brains, roi_values_brain)
```

```
## [1] "working on brain: BADF02 filename:/Volumes/JData/JPeople/Ben/Confocal/chemtag/BADF/nrrds/BADF02-Mz19-GJ851-UAS-CD4-CLIPf_02.nrrd"
## [1] "working on brain: BADF03 filename:/Volumes/JData/JPeople/Ben/Confocal/chemtag/BADF/nrrds/BADF03-Mz19-GJ851-UAS-CD4-CLIPf_02.nrrd"
## [1] "working on brain: BADF05 filename:/Volumes/JData/JPeople/Ben/Confocal/chemtag/BADF/nrrds/BADF05-Mz19-GJ851-UAS-CD4-CLIPf_02.nrrd"
## [1] "working on brain: BADF15 filename:/Volumes/JData/JPeople/Ben/Confocal/chemtag/BADF/nrrds/BADF15-Mz19-GJ851-UAS-CD4-CLIPf_02.nrrd"
## [1] "working on brain: BADH01 filename:/Volumes/JData/JPeople/Ben/Confocal/chemtag/BADH/nrrds/BADH01-mz19-15137-1-2M-UAS-myr-4xCLIPf-VK5_02.nrrd"
## [1] "working on brain: BADH11 filename:/Volumes/JData/JPeople/Ben/Confocal/chemtag/BADH/nrrds/BADH11-mz19-15137-1-2M-UAS-myr-4xCLIPf-VK5_02.nrrd"
## [1] "working on brain: BADH13 filename:/Volumes/JData/JPeople/Ben/Confocal/chemtag/BADH/nrrds/BADH13-mz19-15137-1-2M-UAS-myr-4xCLIPf-VK5_02.nrrd"
## [1] "working on brain: BADH15 filename:/Volumes/JData/JPeople/Ben/Confocal/chemtag/BADH/nrrds/BADH15-mz19-15137-1-2M-UAS-myr-4xCLIPf-VK5_02.nrrd"
## [1] "working on brain: BADS04 filename:/Volumes/JData5/JPeople/Ben/Confocal/chemtag/BADS/nrrds/BADS04_MZ19-15138-1-1M_UAS-myr-4xCLIPf-attP40-CLIP-547_02.nrrd"
## [1] "working on brain: BADS05 filename:/Volumes/JData5/JPeople/Ben/Confocal/chemtag/BADS/nrrds/BADS05_MZ19-15138-1-1M_UAS-myr-4xCLIPf-attP40-CLIP-547_02.nrrd"
## [1] "working on brain: BADS06 filename:/Volumes/JData5/JPeople/Ben/Confocal/chemtag/BADS/nrrds/BADS06_MZ19-15138-1-1M_UAS-myr-4xCLIPf-attP40-CLIP-547_02.nrrd"
## [1] "working on brain: BADS07 filename:/Volumes/JData5/JPeople/Ben/Confocal/chemtag/BADS/nrrds/BADS07_MZ19-15138-1-1M_UAS-myr-4xCLIPf-attP40-CLIP-547_02.nrrd"
## [1] "working on brain: BADS10 filename:/Volumes/JData5/JPeople/Ben/Confocal/chemtag/BADS/nrrds/BADS10_MZ19-GJ853-UAS-CD4-CLIPf-CLIP-547_02.nrrd"
## [1] "working on brain: BADS11 filename:/Volumes/JData5/JPeople/Ben/Confocal/chemtag/BADS/nrrds/BADS11_MZ19-GJ853-UAS-CD4-CLIPf-CLIP-547_02.nrrd"
## [1] "working on brain: BADS13 filename:/Volumes/JData5/JPeople/Ben/Confocal/chemtag/BADS/nrrds/BADS13_MZ19-GJ853-UAS-CD4-CLIPf-CLIP-547_02.nrrd"
## [1] "working on brain: BAER01 filename:/Volumes/JData5/JPeople/Ben/Confocal/chemtag/BAER/nrrds/BAER01_MZ19-GJ838-UAS-myr-SNAPf-attP2-SNAP-549_02.nrrd"
## [1] "working on brain: BAER02 filename:/Volumes/JData5/JPeople/Ben/Confocal/chemtag/BAER/nrrds/BAER02_MZ19-GJ838-UAS-myr-SNAPf-attP2-SNAP-549_02.nrrd"
## [1] "working on brain: BAER03 filename:/Volumes/JData5/JPeople/Ben/Confocal/chemtag/BAER/nrrds/BAER03_MZ19-GJ838-UAS-myr-SNAPf-attP2-SNAP-549_02.nrrd"
## [1] "working on brain: BAER04 filename:/Volumes/JData5/JPeople/Ben/Confocal/chemtag/BAER/nrrds/BAER04_MZ19-GJ838-UAS-myr-SNAPf-attP2-SNAP-549_02.nrrd"
## [1] "working on brain: BAER09 filename:/Volumes/JData5/JPeople/Ben/Confocal/chemtag/BAER/nrrds/BAER09_MZ19-GJ865-UAS-myr-SNAPf-attP40-SNAP-549_02.nrrd"
## [1] "working on brain: BAER10 filename:/Volumes/JData5/JPeople/Ben/Confocal/chemtag/BAER/nrrds/BAER10_MZ19-GJ865-UAS-myr-SNAPf-attP40-SNAP-549_02.nrrd"
## [1] "working on brain: BAER11 filename:/Volumes/JData5/JPeople/Ben/Confocal/chemtag/BAER/nrrds/BAER11_MZ19-GJ865-UAS-myr-SNAPf-attP40-SNAP-549_02.nrrd"
## [1] "working on brain: BAER12 filename:/Volumes/JData5/JPeople/Ben/Confocal/chemtag/BAER/nrrds/BAER12_MZ19-GJ865-UAS-myr-SNAPf-attP40-SNAP-549_02.nrrd"
## [1] "working on brain: BAER13 filename:/Volumes/JData5/JPeople/Ben/Confocal/chemtag/BAER/nrrds/BAER13_MZ19-GJ865-UAS-myr-SNAPf-attP40-SNAP-549_02.nrrd"
## [1] "working on brain: BAER14 filename:/Volumes/JData5/JPeople/Ben/Confocal/chemtag/BAER/nrrds/BAER14_MZ19-15138-2-1M-T07-UAS-myr-4xSNAPf-attP40-SNAP-549_02.nrrd"
## [1] "working on brain: BAER15 filename:/Volumes/JData5/JPeople/Ben/Confocal/chemtag/BAER/nrrds/BAER15_MZ19-15138-2-1M-T07-UAS-myr-4xSNAPf-attP40-SNAP-549_02.nrrd"
## [1] "working on brain: BAER16 filename:/Volumes/JData5/JPeople/Ben/Confocal/chemtag/BAER/nrrds/BAER16_MZ19-15138-2-1M-T07-UAS-myr-4xSNAPf-attP40-SNAP-549_02.nrrd"
## [1] "working on brain: BAER17 filename:/Volumes/JData5/JPeople/Ben/Confocal/chemtag/BAER/nrrds/BAER17_MZ19-15138-2-1M-T07-UAS-myr-4xSNAPf-attP40-SNAP-549_02.nrrd"
## [1] "working on brain: BAER19 filename:/Volumes/JData5/JPeople/Ben/Confocal/chemtag/BAER/nrrds/BAER19_MZ19-15137-2-2M-T02-UAS-myr-4xSNAPf-VK5-SNAP-549_02.nrrd"
## [1] "working on brain: BAER20 filename:/Volumes/JData5/JPeople/Ben/Confocal/chemtag/BAER/nrrds/BAER20_MZ19-15137-2-2M-T02-UAS-myr-4xSNAPf-VK5-SNAP-549_02.nrrd"
## [1] "working on brain: BAER21 filename:/Volumes/JData5/JPeople/Ben/Confocal/chemtag/BAER/nrrds/BAER21_MZ19-15137-2-2M-T02-UAS-myr-4xSNAPf-VK5-SNAP-549_02.nrrd"
## [1] "working on brain: BAER22 filename:/Volumes/JData5/JPeople/Ben/Confocal/chemtag/BAER/nrrds/BAER22_MZ19-15137-2-2M-T02-UAS-myr-4xSNAPf-VK5-SNAP-549_02.nrrd"
## [1] "working on brain: BAER23 filename:/Volumes/JData5/JPeople/Ben/Confocal/chemtag/BAER/nrrds/BAER23_MZ19-15137-2-2M-T02-UAS-myr-4xSNAPf-VK5-SNAP-549_02.nrrd"
## [1] "working on brain: BAFF01 filename:/Volumes/JData5/JPeople/Ben/Confocal/chemtag/BAFF/nrrds/BAFF01_MZ19-UAS-Halo7-CAAX-P40-1_02.nrrd"
## [1] "working on brain: BAFF02 filename:/Volumes/JData5/JPeople/Ben/Confocal/chemtag/BAFF/nrrds/BAFF02_MZ19-UAS-Halo7-CAAX-P40-1_02.nrrd"
## [1] "working on brain: BAFF03 filename:/Volumes/JData5/JPeople/Ben/Confocal/chemtag/BAFF/nrrds/BAFF03_MZ19-UAS-Halo7-CAAX-P40-1_02.nrrd"
## [1] "working on brain: BAFF04 filename:/Volumes/JData5/JPeople/Ben/Confocal/chemtag/BAFF/nrrds/BAFF04_MZ19-UAS-Halo7-CAAX-P40-1_02.nrrd"
## [1] "working on brain: BAFF05 filename:/Volumes/JData5/JPeople/Ben/Confocal/chemtag/BAFF/nrrds/BAFF05_MZ19-UAS-Halo7-CAAX-P40-1_02.nrrd"
## [1] "working on brain: BAFF06 filename:/Volumes/JData5/JPeople/Ben/Confocal/chemtag/BAFF/nrrds/BAFF06_MZ19-UAS-Halo7-CAAX-P40-1_02.nrrd"
## [1] "working on brain: BAFF07 filename:/Volumes/JData5/JPeople/Ben/Confocal/chemtag/BAFF/nrrds/BAFF07_MZ19-UAS-Halo7-CAAX-P40-1_02.nrrd"
## [1] "working on brain: BAFF08 filename:/Volumes/JData5/JPeople/Ben/Confocal/chemtag/BAFF/nrrds/BAFF08_MZ19-UAS-td3-Halo7-CAAX-P40-2_02.nrrd"
## [1] "working on brain: BAFF09 filename:/Volumes/JData5/JPeople/Ben/Confocal/chemtag/BAFF/nrrds/BAFF09_MZ19-UAS-td3-Halo7-CAAX-P40-2_02.nrrd"
## [1] "working on brain: BAFF10 filename:/Volumes/JData5/JPeople/Ben/Confocal/chemtag/BAFF/nrrds/BAFF10_MZ19-UAS-td3-Halo7-CAAX-P40-2_02.nrrd"
## [1] "working on brain: BAFF11 filename:/Volumes/JData5/JPeople/Ben/Confocal/chemtag/BAFF/nrrds/BAFF11_MZ19-UAS-td3-Halo7-CAAX-P40-2_02.nrrd"
## [1] "working on brain: BAFF12 filename:/Volumes/JData5/JPeople/Ben/Confocal/chemtag/BAFF/nrrds/BAFF12_MZ19-UAS-td3-Halo7-CAAX-P40-2_02.nrrd"
## [1] "working on brain: BAFF13 filename:/Volumes/JData5/JPeople/Ben/Confocal/chemtag/BAFF/nrrds/BAFF13_MZ19-UAS-td3-Halo7-CAAX-P40-2_02.nrrd"
## [1] "working on brain: BAFF14 filename:/Volumes/JData5/JPeople/Ben/Confocal/chemtag/BAFF/nrrds/BAFF14_MZ19-UAS-td3-Halo7-CAAX-P40-2_02.nrrd"
## [1] "working on brain: BAFF15 filename:/Volumes/JData5/JPeople/Ben/Confocal/chemtag/BAFF/nrrds/BAFF15_MZ19-UAS-td3-Halo7-CAAX-P40-2_02.nrrd"
## [1] "working on brain: BAFF16 filename:/Volumes/JData5/JPeople/Ben/Confocal/chemtag/BAFF/nrrds/BAFF16_MZ19-UAS-td7-Halo7-CAAX-P40-1_02.nrrd"
## [1] "working on brain: BAFF17 filename:/Volumes/JData5/JPeople/Ben/Confocal/chemtag/BAFF/nrrds/BAFF17_MZ19-UAS-td7-Halo7-CAAX-P40-1_02.nrrd"
## [1] "working on brain: BAFF18 filename:/Volumes/JData5/JPeople/Ben/Confocal/chemtag/BAFF/nrrds/BAFF18_MZ19-UAS-td7-Halo7-CAAX-P40-1_02.nrrd"
## [1] "working on brain: BAFF19 filename:/Volumes/JData5/JPeople/Ben/Confocal/chemtag/BAFF/nrrds/BAFF19_MZ19-UAS-td7-Halo7-CAAX-P40-1_02.nrrd"
## [1] "working on brain: BAFF20 filename:/Volumes/JData5/JPeople/Ben/Confocal/chemtag/BAFF/nrrds/BAFF20_MZ19-UAS-td7-Halo7-CAAX-P40-1_02.nrrd"
## [1] "working on brain: BAFF21 filename:/Volumes/JData5/JPeople/Ben/Confocal/chemtag/BAFF/nrrds/BAFF21_MZ19-UAS-td7-Halo7-CAAX-P40-1_02.nrrd"
## [1] "working on brain: BAFF22 filename:/Volumes/JData5/JPeople/Ben/Confocal/chemtag/BAFF/nrrds/BAFF22_MZ19-UAS-td7-Halo7-CAAX-P40-1_02.nrrd"
## [1] "working on brain: BAFF23 filename:/Volumes/JData5/JPeople/Ben/Confocal/chemtag/BAFF/nrrds/BAFF23_MZ19-UAS-td7-Halo7-CAAX-P40-1_02.nrrd"
## [1] "working on brain: BAFF24 filename:/Volumes/JData5/JPeople/Ben/Confocal/chemtag/BAFF/nrrds/BAFF24_MZ19-UAS-myr-Halo2-P40_02.nrrd"
## [1] "working on brain: BAFF25 filename:/Volumes/JData5/JPeople/Ben/Confocal/chemtag/BAFF/nrrds/BAFF25_MZ19-UAS-myr-Halo2-P40_02.nrrd"
## [1] "working on brain: BAFF26 filename:/Volumes/JData5/JPeople/Ben/Confocal/chemtag/BAFF/nrrds/BAFF26_MZ19-UAS-myr-Halo2-P40_02.nrrd"
## [1] "working on brain: BAFF27 filename:/Volumes/JData5/JPeople/Ben/Confocal/chemtag/BAFF/nrrds/BAFF27_MZ19-UAS-myr-Halo2-P40_02.nrrd"
## [1] "working on brain: BAFF28 filename:/Volumes/JData5/JPeople/Ben/Confocal/chemtag/BAFF/nrrds/BAFF28_MZ19-UAS-myr-Halo2-P40_02.nrrd"
## [1] "working on brain: BAFF29 filename:/Volumes/JData5/JPeople/Ben/Confocal/chemtag/BAFF/nrrds/BAFF29_MZ19-UAS-myr-Halo2-P40_02.nrrd"
## [1] "working on brain: BAFF30 filename:/Volumes/JData5/JPeople/Ben/Confocal/chemtag/BAFF/nrrds/BAFF30_MZ19-UAS-myr-Halo2-P40_02.nrrd"
```

Turn results into a data.frame

```
brain_results_df=do.call(rbind, brain_results)
brain_results_df$fg.bsub=brain_results_df$foreground-brain_results_df$background
brain_results_df$name=sub('VAd1','VA1d', brain_results_df$name)
# fix level 
brain_results_df=droplevels(brain_results_df)
brain_results_df$region=sub("^[LR]","",brain_results_df$name)
kable(brain_results_df)
```

|  | name | id | foreground | background | brain | fg.bsub | region |
| --- | --- | --- | --- | --- | --- | --- | --- |
| 3 | LVA1d | 3 | NaN | NaN | BADF02 | NaN | VA1d |
| 4 | RLH | 4 | 843.2256 | 355.63275 | BADF02 | 487.5928 | LH |
| 5 | RCB | 5 | NaN | NaN | BADF02 | NaN | CB |
| 31 | LVA1d | 3 | NaN | NaN | BADF03 | NaN | VA1d |
| 41 | RLH | 4 | 903.7537 | 479.44002 | BADF03 | 424.3137 | LH |
| 51 | RCB | 5 | NaN | NaN | BADF03 | NaN | CB |
| 32 | LVA1d | 3 | NaN | NaN | BADF05 | NaN | VA1d |
| 42 | RLH | 4 | 831.9586 | 435.47620 | BADF05 | 396.4824 | LH |
| 52 | RCB | 5 | NaN | NaN | BADF05 | NaN | CB |
| 33 | LVA1d | 3 | NaN | NaN | BADF15 | NaN | VA1d |
| 43 | RLH | 4 | 647.7242 | 345.90502 | BADF15 | 301.8191 | LH |
| 53 | RCB | 5 | NaN | NaN | BADF15 | NaN | CB |
| 34 | LVA1d | 3 | NaN | NaN | BADH01 | NaN | VA1d |
| 44 | RLH | 4 | 17703.0015 | 749.47485 | BADH01 | 16953.5267 | LH |
| 54 | RCB | 5 | NaN | NaN | BADH01 | NaN | CB |
| 35 | LVA1d | 3 | NaN | NaN | BADH11 | NaN | VA1d |
| 45 | RLH | 4 | 11693.1782 | 489.64485 | BADH11 | 11203.5333 | LH |
| 55 | RCB | 5 | NaN | NaN | BADH11 | NaN | CB |
| 36 | LVA1d | 3 | NaN | NaN | BADH13 | NaN | VA1d |
| 46 | RLH | 4 | 12008.2871 | 463.85109 | BADH13 | 11544.4360 | LH |
| 56 | RCB | 5 | NaN | NaN | BADH13 | NaN | CB |
| 37 | LVA1d | 3 | NaN | NaN | BADH15 | NaN | VA1d |
| 47 | RLH | 4 | 11130.8039 | 489.61116 | BADH15 | 10641.1928 | LH |
| 57 | RCB | 5 | NaN | NaN | BADH15 | NaN | CB |
| 38 | LVA1d | 3 | 65356.4140 | 3683.23175 | BADS04 | 61673.1823 | VA1d |
| 48 | RLH | 4 | 34953.6490 | 2187.74807 | BADS04 | 32765.9009 | LH |
| 58 | RCB | 5 | 33068.9400 | 3570.30037 | BADS04 | 29498.6397 | CB |
| 39 | LVA1d | 3 | 61861.9038 | 4289.85410 | BADS05 | 57572.0497 | VA1d |
| 49 | RLH | 4 | 27734.9618 | 2639.60192 | BADS05 | 25095.3599 | LH |
| 59 | RCB | 5 | 44376.4191 | 1824.57507 | BADS05 | 42551.8440 | CB |
| 310 | LVA1d | 3 | 62219.7081 | 8014.38805 | BADS06 | 54205.3201 | VA1d |
| 410 | RLH | 4 | 40600.6447 | 3003.43628 | BADS06 | 37597.2084 | LH |
| 510 | RCB | 5 | 44316.5037 | 5851.93053 | BADS06 | 38464.5732 | CB |
| 311 | LVA1d | 3 | 65533.1919 | 9450.46209 | BADS07 | 56082.7299 | VA1d |
| 411 | RLH | 4 | 42306.2329 | 1706.53698 | BADS07 | 40599.6959 | LH |
| 511 | RCB | 5 | 41531.6441 | 1904.49593 | BADS07 | 39627.1482 | CB |
| 312 | LVA1d | 3 | 4144.4893 | 2045.57194 | BADS10 | 2098.9174 | VA1d |
| 412 | RLH | 4 | 1433.8164 | 650.32322 | BADS10 | 783.4932 | LH |
| 512 | RCB | 5 | 2277.9127 | 803.04727 | BADS10 | 1474.8654 | CB |
| 313 | LVA1d | 3 | 3240.3620 | 1776.68776 | BADS11 | 1463.6742 | VA1d |
| 413 | RLH | 4 | 1851.5378 | 851.22912 | BADS11 | 1000.3087 | LH |
| 513 | RCB | 5 | 3197.5722 | 1915.63817 | BADS11 | 1281.9341 | CB |
| 314 | LVA1d | 3 | 3894.5959 | 2002.58070 | BADS13 | 1892.0152 | VA1d |
| 414 | RLH | 4 | 689.4990 | 476.70310 | BADS13 | 212.7959 | LH |
| 514 | LCB | 5 | 2876.6060 | 959.95729 | BADS13 | 1916.6487 | CB |
| 315 | LVA1d | 3 | 1850.7775 | 587.79448 | BAER01 | 1262.9830 | VA1d |
| 415 | RLH | 4 | 1188.3191 | 149.02348 | BAER01 | 1039.2956 | LH |
| 515 | RCB | 5 | 935.3903 | 311.35748 | BAER01 | 624.0328 | CB |
| 316 | LVA1d | 3 | 2140.7128 | 427.44068 | BAER02 | 1713.2721 | VA1d |
| 416 | RLH | 4 | 1186.4472 | 161.04012 | BAER02 | 1025.4071 | LH |
| 516 | RCB | 5 | 984.3606 | 279.58134 | BAER02 | 704.7792 | CB |
| 317 | LVA1d | 3 | 1815.4736 | 609.35763 | BAER03 | 1206.1160 | VA1d |
| 417 | RLH | 4 | 915.8943 | 121.96713 | BAER03 | 793.9271 | LH |
| 517 | RCB | 5 | 1157.2331 | 341.73046 | BAER03 | 815.5026 | CB |
| 318 | LVA1d | 3 | 1716.1167 | 450.47742 | BAER04 | 1265.6393 | VA1d |
| 418 | RLH | 4 | 937.2071 | 132.46147 | BAER04 | 804.7456 | LH |
| 518 | RCB | 5 | 1164.5871 | 329.36201 | BAER04 | 835.2251 | CB |
| 319 | LVA1d | 3 | 2557.9007 | 432.39365 | BAER09 | 2125.5070 | VA1d |
| 419 | RLH | 4 | 1637.0325 | 154.18360 | BAER09 | 1482.8489 | LH |
| 519 | RCB | 5 | 1235.8822 | 326.31057 | BAER09 | 909.5717 | CB |
| 320 | LVA1d | 3 | 3454.5415 | 461.92185 | BAER10 | 2992.6197 | VA1d |
| 420 | RLH | 4 | 1777.8672 | 173.63089 | BAER10 | 1604.2364 | LH |
| 520 | RCB | 5 | 1079.2446 | 270.05591 | BAER10 | 809.1887 | CB |
| 321 | LVA1d | 3 | 2777.7459 | 576.19742 | BAER11 | 2201.5484 | VA1d |
| 421 | RLH | 4 | 2798.3732 | 216.41141 | BAER11 | 2581.9618 | LH |
| 521 | RCB | 5 | 1912.5392 | 426.31805 | BAER11 | 1486.2212 | CB |
| 322 | LVA1d | 3 | 4100.6239 | 392.52469 | BAER12 | 3708.0992 | VA1d |
| 422 | RLH | 4 | 1479.9117 | 156.80587 | BAER12 | 1323.1058 | LH |
| 522 | RCB | 5 | 1320.8687 | 270.51249 | BAER12 | 1050.3562 | CB |
| 323 | LVA1d | 3 | 4180.1720 | 691.12247 | BAER13 | 3489.0495 | VA1d |
| 423 | RLH | 4 | 1740.9918 | 231.94303 | BAER13 | 1509.0488 | LH |
| 523 | RCB | 5 | 1686.5253 | 639.21161 | BAER13 | 1047.3136 | CB |
| 324 | LVA1d | 3 | 47541.9688 | 1812.90241 | BAER14 | 45729.0664 | VA1d |
| 424 | RLH | 4 | 10363.6268 | 591.00025 | BAER14 | 9772.6266 | LH |
| 524 | RCB | 5 | 14226.1697 | 588.63568 | BAER14 | 13637.5341 | CB |
| 325 | LVA1d | 3 | 40436.8522 | 1480.78080 | BAER15 | 38956.0714 | VA1d |
| 425 | RLH | 4 | 7501.7214 | 414.60371 | BAER15 | 7087.1177 | LH |
| 525 | RCB | 5 | 14710.5902 | 770.73167 | BAER15 | 13939.8585 | CB |
| 326 | LVA1d | 3 | 37120.9514 | 1250.30669 | BAER16 | 35870.6448 | VA1d |
| 426 | RLH | 4 | 9494.6148 | 390.02992 | BAER16 | 9104.5849 | LH |
| 526 | RCB | 5 | 28621.7306 | 728.03415 | BAER16 | 27893.6964 | CB |
| 327 | LVA1d | 3 | 41810.0092 | 350.06246 | BAER17 | 41459.9467 | VA1d |
| 427 | RLH | 4 | 8995.6560 | 452.69617 | BAER17 | 8542.9599 | LH |
| 527 | RCB | 5 | 14701.2771 | 523.49263 | BAER17 | 14177.7844 | CB |
| 328 | LVA1d | 3 | 38527.8392 | 1213.66731 | BAER19 | 37314.1719 | VA1d |
| 428 | RLH | 4 | 11022.7959 | 468.80105 | BAER19 | 10553.9949 | LH |
| 528 | RCB | 5 | 17447.7442 | 970.73344 | BAER19 | 16477.0108 | CB |
| 329 | LVA1d | 3 | 43497.4014 | 2287.28193 | BAER20 | 41210.1194 | VA1d |
| 429 | RLH | 4 | 14711.7687 | 414.22749 | BAER20 | 14297.5413 | LH |
| 529 | RCB | 5 | 18146.9206 | 1488.29280 | BAER20 | 16658.6278 | CB |
| 330 | LVA1d | 3 | 35395.9744 | 1749.71662 | BAER21 | 33646.2578 | VA1d |
| 430 | RLH | 4 | 14182.0832 | 489.58915 | BAER21 | 13692.4941 | LH |
| 530 | RCB | 5 | 13614.7491 | 689.08047 | BAER21 | 12925.6686 | CB |
| 331 | LVA1d | 3 | 37171.3005 | 2984.96666 | BAER22 | 34186.3338 | VA1d |
| 431 | RLH | 4 | 14362.4262 | 361.35797 | BAER22 | 14001.0682 | LH |
| 531 | RCB | 5 | 13470.3080 | 719.15454 | BAER22 | 12751.1535 | CB |
| 332 | LVA1d | 3 | 41902.7590 | 1592.96515 | BAER23 | 40309.7938 | VA1d |
| 432 | RLH | 4 | 11011.0558 | 427.63576 | BAER23 | 10583.4201 | LH |
| 532 | RCB | 5 | 11011.6017 | 870.56500 | BAER23 | 10141.0367 | CB |
| 333 | LVA1d | 3 | NaN | NaN | BAFF01 | NaN | VA1d |
| 433 | RLH | 4 | 390.9094 | 68.39040 | BAFF01 | 322.5190 | LH |
| 533 | RCB | 5 | NaN | NaN | BAFF01 | NaN | CB |
| 334 | LVA1d | 3 | NaN | NaN | BAFF02 | NaN | VA1d |
| 434 | RLH | 4 | 372.1502 | 65.75094 | BAFF02 | 306.3992 | LH |
| 534 | RCB | 5 | NaN | NaN | BAFF02 | NaN | CB |
| 335 | LVA1d | 3 | NaN | NaN | BAFF03 | NaN | VA1d |
| 435 | RLH | 4 | 418.7782 | 83.59495 | BAFF03 | 335.1833 | LH |
| 535 | RCB | 5 | NaN | NaN | BAFF03 | NaN | CB |
| 336 | LVA1d | 3 | NaN | NaN | BAFF04 | NaN | VA1d |
| 436 | RLH | 4 | 338.5232 | 75.40786 | BAFF04 | 263.1153 | LH |
| 536 | RCB | 5 | NaN | NaN | BAFF04 | NaN | CB |
| 337 | LVA1d | 3 | NaN | NaN | BAFF05 | NaN | VA1d |
| 437 | RLH | 4 | 765.7880 | 68.71237 | BAFF05 | 697.0756 | LH |
| 537 | RCB | 5 | NaN | NaN | BAFF05 | NaN | CB |
| 338 | LVA1d | 3 | NaN | NaN | BAFF06 | NaN | VA1d |
| 438 | RLH | 4 | 378.8666 | 66.89089 | BAFF06 | 311.9757 | LH |
| 538 | RCB | 5 | NaN | NaN | BAFF06 | NaN | CB |
| 339 | LVA1d | 3 | NaN | NaN | BAFF07 | NaN | VA1d |
| 439 | RLH | 4 | 314.6876 | 83.11179 | BAFF07 | 231.5758 | LH |
| 539 | RCB | 5 | NaN | NaN | BAFF07 | NaN | CB |
| 340 | LVA1d | 3 | NaN | NaN | BAFF08 | NaN | VA1d |
| 440 | RLH | 4 | 516.5100 | 102.41340 | BAFF08 | 414.0966 | LH |
| 540 | RCB | 5 | NaN | NaN | BAFF08 | NaN | CB |
| 341 | LVA1d | 3 | NaN | NaN | BAFF09 | NaN | VA1d |
| 441 | RLH | 4 | 528.0184 | 86.07839 | BAFF09 | 441.9400 | LH |
| 541 | RCB | 5 | NaN | NaN | BAFF09 | NaN | CB |
| 342 | LVA1d | 3 | NaN | NaN | BAFF10 | NaN | VA1d |
| 442 | RLH | 4 | 551.7946 | 111.35376 | BAFF10 | 440.4409 | LH |
| 542 | RCB | 5 | NaN | NaN | BAFF10 | NaN | CB |
| 343 | LVA1d | 3 | NaN | NaN | BAFF11 | NaN | VA1d |
| 443 | RLH | 4 | 543.0926 | 87.04913 | BAFF11 | 456.0435 | LH |
| 543 | RCB | 5 | NaN | NaN | BAFF11 | NaN | CB |
| 344 | LVA1d | 3 | NaN | NaN | BAFF12 | NaN | VA1d |
| 444 | RLH | 4 | 342.0960 | 65.19292 | BAFF12 | 276.9030 | LH |
| 544 | RCB | 5 | NaN | NaN | BAFF12 | NaN | CB |
| 345 | LVA1d | 3 | NaN | NaN | BAFF13 | NaN | VA1d |
| 445 | RLH | 4 | 468.0527 | 87.39650 | BAFF13 | 380.6562 | LH |
| 545 | RCB | 5 | NaN | NaN | BAFF13 | NaN | CB |
| 346 | LVA1d | 3 | NaN | NaN | BAFF14 | NaN | VA1d |
| 446 | RLH | 4 | 519.5695 | 102.73657 | BAFF14 | 416.8330 | LH |
| 546 | RCB | 5 | NaN | NaN | BAFF14 | NaN | CB |
| 347 | LVA1d | 3 | NaN | NaN | BAFF15 | NaN | VA1d |
| 447 | RLH | 4 | 563.3779 | 105.41412 | BAFF15 | 457.9638 | LH |
| 547 | RCB | 5 | NaN | NaN | BAFF15 | NaN | CB |
| 348 | LVA1d | 3 | NaN | NaN | BAFF16 | NaN | VA1d |
| 448 | RLH | 4 | 201.3274 | 44.01812 | BAFF16 | 157.3093 | LH |
| 548 | RCB | 5 | NaN | NaN | BAFF16 | NaN | CB |
| 349 | LVA1d | 3 | NaN | NaN | BAFF17 | NaN | VA1d |
| 449 | RLH | 4 | 286.3674 | 64.19660 | BAFF17 | 222.1708 | LH |
| 549 | RCB | 5 | NaN | NaN | BAFF17 | NaN | CB |
| 350 | LVA1d | 3 | NaN | NaN | BAFF18 | NaN | VA1d |
| 450 | RLH | 4 | 271.9601 | 61.75146 | BAFF18 | 210.2087 | LH |
| 550 | RCB | 5 | NaN | NaN | BAFF18 | NaN | CB |
| 351 | VLA1d | 3 | NaN | NaN | BAFF19 | NaN | VLA1d |
| 451 | RLH | 4 | 227.8673 | 46.11702 | BAFF19 | 181.7503 | LH |
| 551 | RCB | 5 | NaN | NaN | BAFF19 | NaN | CB |
| 352 | VLA1d | 3 | NaN | NaN | BAFF20 | NaN | VLA1d |
| 452 | RLH | 4 | 142.5434 | 28.58156 | BAFF20 | 113.9618 | LH |
| 552 | RCB | 5 | NaN | NaN | BAFF20 | NaN | CB |
| 353 | LVA1d | 3 | NaN | NaN | BAFF21 | NaN | VA1d |
| 453 | RLH | 4 | 192.9050 | 49.71022 | BAFF21 | 143.1947 | LH |
| 553 | RCB | 5 | NaN | NaN | BAFF21 | NaN | CB |
| 354 | LVA1d | 3 | NaN | NaN | BAFF22 | NaN | VA1d |
| 454 | RLH | 4 | 178.6128 | 59.01869 | BAFF22 | 119.5942 | LH |
| 554 | RCB | 5 | NaN | NaN | BAFF22 | NaN | CB |
| 355 | LVA1d | 3 | NaN | NaN | BAFF23 | NaN | VA1d |
| 455 | RLH | 4 | 236.7702 | 50.95907 | BAFF23 | 185.8111 | LH |
| 555 | RCB | 5 | NaN | NaN | BAFF23 | NaN | CB |
| 356 | LVA1d | 3 | NaN | NaN | BAFF24 | NaN | VA1d |
| 456 | RLH | 4 | 310.6939 | 64.75611 | BAFF24 | 245.9377 | LH |
| 556 | RCB | 5 | NaN | NaN | BAFF24 | NaN | CB |
| 357 | LVA1d | 3 | NaN | NaN | BAFF25 | NaN | VA1d |
| 457 | RLH | 4 | 250.0917 | 46.05495 | BAFF25 | 204.0368 | LH |
| 557 | RCB | 5 | NaN | NaN | BAFF25 | NaN | CB |
| 358 | LVA1d | 3 | NaN | NaN | BAFF26 | NaN | VA1d |
| 458 | RLH | 4 | 257.4146 | 50.51325 | BAFF26 | 206.9014 | LH |
| 558 | RCB | 5 | NaN | NaN | BAFF26 | NaN | CB |
| 359 | LVA1d | 3 | NaN | NaN | BAFF27 | NaN | VA1d |
| 459 | RLH | 4 | 298.6770 | 51.77979 | BAFF27 | 246.8972 | LH |
| 559 | RCB | 5 | NaN | NaN | BAFF27 | NaN | CB |
| 360 | LVA1d | 3 | NaN | NaN | BAFF28 | NaN | VA1d |
| 460 | RLH | 4 | 271.8469 | 48.49441 | BAFF28 | 223.3525 | LH |
| 560 | RCB | 5 | NaN | NaN | BAFF28 | NaN | CB |
| 361 | LVA1d | 3 | NaN | NaN | BAFF29 | NaN | VA1d |
| 461 | RLH | 4 | 314.4700 | 55.96135 | BAFF29 | 258.5086 | LH |
| 561 | RCB | 5 | NaN | NaN | BAFF29 | NaN | CB |
| 362 | LVA1d | 3 | NaN | NaN | BAFF30 | NaN | VA1d |
| 462 | RLH | 4 | 282.4809 | 51.66062 | BAFF30 | 230.8202 | LH |
| 562 | RCB | 5 | NaN | NaN | BAFF30 | NaN | CB |

```
write.csv(brain_results_df, file='brain_results_df.csv')
```

```
library(ggplot2)
m=merge(brain_results_df, braindf, by='brain')
lp <- qplot(data=m, reporter, fg.bsub, facets= . ~ region, geom='boxplot', col=reporter) +scale_y_log10(breaks=c(30,100,300,1000,3e3,10e3,30e3),labels=c(30,100,300,1000,3e3,10e3,30e3))
lp <- lp+ylab('Intensity /au')
#lp+ scale_colour_manual(values = c('CD4-CLIPf'='grey', 'myr-4xCLIPf'='red', 'myr-4xSNAPf'='cyan', 'myr-SNAPf'='blue'), labels=c("old","new", "new", "old"))
lp+annotation_logticks(sides='l')
```

```
## Warning: Removed 76 rows containing non-finite values (stat_boxplot).
```

```
qplot(data=m, reporter, fg.bsub, facets= . ~ region, geom='boxplot')
```

```
## Warning: Removed 76 rows containing non-finite values (stat_boxplot).
```

```
library(dplyr)
```

```
## 
## Attaching package: 'dplyr'
```

```
## The following objects are masked from 'package:nat':
## 
##     intersect, setdiff, union
```

```
## The following objects are masked from 'package:stats':
## 
##     filter, lag
```

```
## The following objects are masked from 'package:base':
## 
##     intersect, setdiff, setequal, union
```

```
gm=m %>% group_by(region, reporter, insertion) %>%
  summarise(val=mean(fg.bsub))
kable(gm)
```

| region | reporter | insertion | val |
| --- | --- | --- | --- |
| CB | CD4-CLIPf | GJ851 | NaN |
| CB | CD4-CLIPf | GJ853 | 1557.8161 |
| CB | myr-4xCLIPf | P40 | 37535.5513 |
| CB | myr-4xCLIPf | VK5 | NaN |
| CB | myr-4xSNAPf | P40 | 17412.2184 |
| CB | myr-4xSNAPf | VK5 | 13790.6995 |
| CB | myr-Halo2 | P40 | NaN |
| CB | myr-SNAPf | P2 | 744.8850 |
| CB | myr-SNAPf | P40 | 1060.5303 |
| CB | UAS-Halo7-CAAX | P40 | NaN |
| CB | UAS-td3-Halo7-CAAX | P40 | NaN |
| CB | UAS-td7-Halo7-CAAX | P40 | NaN |
| LH | CD4-CLIPf | GJ851 | 402.5520 |
| LH | CD4-CLIPf | GJ853 | 665.5326 |
| LH | myr-4xCLIPf | P40 | 34014.5413 |
| LH | myr-4xCLIPf | VK5 | 12585.6722 |
| LH | myr-4xSNAPf | P40 | 8626.8223 |
| LH | myr-4xSNAPf | VK5 | 12625.7037 |
| LH | myr-Halo2 | P40 | 230.9221 |
| LH | myr-SNAPf | P2 | 915.8439 |
| LH | myr-SNAPf | P40 | 1700.2403 |
| LH | UAS-Halo7-CAAX | P40 | 352.5491 |
| LH | UAS-td3-Halo7-CAAX | P40 | 410.6096 |
| LH | UAS-td7-Halo7-CAAX | P40 | 166.7501 |
| VA1d | CD4-CLIPf | GJ851 | NaN |
| VA1d | CD4-CLIPf | GJ853 | 1818.2023 |
| VA1d | myr-4xCLIPf | P40 | 57383.3205 |
| VA1d | myr-4xCLIPf | VK5 | NaN |
| VA1d | myr-4xSNAPf | P40 | 40503.9323 |
| VA1d | myr-4xSNAPf | VK5 | 37333.3354 |
| VA1d | myr-Halo2 | P40 | NaN |
| VA1d | myr-SNAPf | P2 | 1362.0026 |
| VA1d | myr-SNAPf | P40 | 2903.3648 |
| VA1d | UAS-Halo7-CAAX | P40 | NaN |
| VA1d | UAS-td3-Halo7-CAAX | P40 | NaN |
| VA1d | UAS-td7-Halo7-CAAX | P40 | NaN |
| VLA1d | UAS-td7-Halo7-CAAX | P40 | NaN |

```
gm$val=round(gm$val, 0)
write.csv(gm[gm$region=="LH",c('reporter', 'insertion','val')], file='LH_results.csv')
write
```

```
## function (x, file = "data", ncolumns = if (is.character(x)) 1 else 5, 
##     append = FALSE, sep = " ") 
## cat(x, file = file, sep = c(rep.int(sep, ncolumns - 1), "\n"), 
##     append = append)
## <bytecode: 0x7f9cb511fc68>
## <environment: namespace:base>
```

```
# Seba experimenting with SNAP plots
mSNAPlh=subset(m[grep("SNAP",m$reporter),], region == "LH")
# Next we need to specify the order of the levels to get the plot in the right order
mSNAPlh$reporter = factor(mSNAPlh$reporter,levels=c("CD4-CLIPf","myr-4xCLIPf","myr-SNAPf","myr-4xSNAPf"))
kable(mSNAPlh)
```

|  | brain | name | id | foreground | background | fg.bsub | region | low | driver | reporter | insertion |
| --- | --- | --- | --- | --- | --- | --- | --- | --- | --- | --- | --- |
| 47 | BAER01 | RLH | 4 | 1188.3191 | 149.0235 | 1039.2956 | LH | /Volumes/JData5/JPeople/Ben/Confocal/chemtag/BAER/nrrds/BAER01\_MZ19-GJ838-UAS-myr-SNAPf-attP2-SNAP-549\_02.nrrd | MZ19 | myr-SNAPf | P2 |
| 50 | BAER02 | RLH | 4 | 1186.4472 | 161.0401 | 1025.4071 | LH | /Volumes/JData5/JPeople/Ben/Confocal/chemtag/BAER/nrrds/BAER02\_MZ19-GJ838-UAS-myr-SNAPf-attP2-SNAP-549\_02.nrrd | MZ19 | myr-SNAPf | P2 |
| 53 | BAER03 | RLH | 4 | 915.8943 | 121.9671 | 793.9271 | LH | /Volumes/JData5/JPeople/Ben/Confocal/chemtag/BAER/nrrds/BAER03\_MZ19-GJ838-UAS-myr-SNAPf-attP2-SNAP-549\_02.nrrd | MZ19 | myr-SNAPf | P2 |
| 56 | BAER04 | RLH | 4 | 937.2071 | 132.4615 | 804.7456 | LH | /Volumes/JData5/JPeople/Ben/Confocal/chemtag/BAER/nrrds/BAER04\_MZ19-GJ838-UAS-myr-SNAPf-attP2-SNAP-549\_02.nrrd | MZ19 | myr-SNAPf | P2 |
| 59 | BAER09 | RLH | 4 | 1637.0325 | 154.1836 | 1482.8489 | LH | /Volumes/JData5/JPeople/Ben/Confocal/chemtag/BAER/nrrds/BAER09\_MZ19-GJ865-UAS-myr-SNAPf-attP40-SNAP-549\_02.nrrd | MZ19 | myr-SNAPf | P40 |
| 62 | BAER10 | RLH | 4 | 1777.8672 | 173.6309 | 1604.2364 | LH | /Volumes/JData5/JPeople/Ben/Confocal/chemtag/BAER/nrrds/BAER10\_MZ19-GJ865-UAS-myr-SNAPf-attP40-SNAP-549\_02.nrrd | MZ19 | myr-SNAPf | P40 |
| 65 | BAER11 | RLH | 4 | 2798.3732 | 216.4114 | 2581.9618 | LH | /Volumes/JData5/JPeople/Ben/Confocal/chemtag/BAER/nrrds/BAER11\_MZ19-GJ865-UAS-myr-SNAPf-attP40-SNAP-549\_02.nrrd | MZ19 | myr-SNAPf | P40 |
| 68 | BAER12 | RLH | 4 | 1479.9117 | 156.8059 | 1323.1058 | LH | /Volumes/JData5/JPeople/Ben/Confocal/chemtag/BAER/nrrds/BAER12\_MZ19-GJ865-UAS-myr-SNAPf-attP40-SNAP-549\_02.nrrd | MZ19 | myr-SNAPf | P40 |
| 71 | BAER13 | RLH | 4 | 1740.9918 | 231.9430 | 1509.0488 | LH | /Volumes/JData5/JPeople/Ben/Confocal/chemtag/BAER/nrrds/BAER13\_MZ19-GJ865-UAS-myr-SNAPf-attP40-SNAP-549\_02.nrrd | MZ19 | myr-SNAPf | P40 |
| 74 | BAER14 | RLH | 4 | 10363.6268 | 591.0003 | 9772.6266 | LH | /Volumes/JData5/JPeople/Ben/Confocal/chemtag/BAER/nrrds/BAER14\_MZ19-15138-2-1M-T07-UAS-myr-4xSNAPf-attP40-SNAP-549\_02.nrrd | MZ19 | myr-4xSNAPf | P40 |
| 77 | BAER15 | RLH | 4 | 7501.7214 | 414.6037 | 7087.1177 | LH | /Volumes/JData5/JPeople/Ben/Confocal/chemtag/BAER/nrrds/BAER15\_MZ19-15138-2-1M-T07-UAS-myr-4xSNAPf-attP40-SNAP-549\_02.nrrd | MZ19 | myr-4xSNAPf | P40 |
| 80 | BAER16 | RLH | 4 | 9494.6148 | 390.0299 | 9104.5849 | LH | /Volumes/JData5/JPeople/Ben/Confocal/chemtag/BAER/nrrds/BAER16\_MZ19-15138-2-1M-T07-UAS-myr-4xSNAPf-attP40-SNAP-549\_02.nrrd | MZ19 | myr-4xSNAPf | P40 |
| 83 | BAER17 | RLH | 4 | 8995.6560 | 452.6962 | 8542.9599 | LH | /Volumes/JData5/JPeople/Ben/Confocal/chemtag/BAER/nrrds/BAER17\_MZ19-15138-2-1M-T07-UAS-myr-4xSNAPf-attP40-SNAP-549\_02.nrrd | MZ19 | myr-4xSNAPf | P40 |
| 86 | BAER19 | RLH | 4 | 11022.7959 | 468.8011 | 10553.9949 | LH | /Volumes/JData5/JPeople/Ben/Confocal/chemtag/BAER/nrrds/BAER19\_MZ19-15137-2-2M-T02-UAS-myr-4xSNAPf-VK5-SNAP-549\_02.nrrd | MZ19 | myr-4xSNAPf | VK5 |
| 89 | BAER20 | RLH | 4 | 14711.7687 | 414.2275 | 14297.5413 | LH | /Volumes/JData5/JPeople/Ben/Confocal/chemtag/BAER/nrrds/BAER20\_MZ19-15137-2-2M-T02-UAS-myr-4xSNAPf-VK5-SNAP-549\_02.nrrd | MZ19 | myr-4xSNAPf | VK5 |
| 92 | BAER21 | RLH | 4 | 14182.0832 | 489.5891 | 13692.4941 | LH | /Volumes/JData5/JPeople/Ben/Confocal/chemtag/BAER/nrrds/BAER21\_MZ19-15137-2-2M-T02-UAS-myr-4xSNAPf-VK5-SNAP-549\_02.nrrd | MZ19 | myr-4xSNAPf | VK5 |
| 95 | BAER22 | RLH | 4 | 14362.4262 | 361.3580 | 14001.0682 | LH | /Volumes/JData5/JPeople/Ben/Confocal/chemtag/BAER/nrrds/BAER22\_MZ19-15137-2-2M-T02-UAS-myr-4xSNAPf-VK5-SNAP-549\_02.nrrd | MZ19 | myr-4xSNAPf | VK5 |
| 98 | BAER23 | RLH | 4 | 11011.0558 | 427.6358 | 10583.4201 | LH | /Volumes/JData5/JPeople/Ben/Confocal/chemtag/BAER/nrrds/BAER23\_MZ19-15137-2-2M-T02-UAS-myr-4xSNAPf-VK5-SNAP-549\_02.nrrd | MZ19 | myr-4xSNAPf | VK5 |

```
lp <- qplot(data=mSNAPlh, insertion, fg.bsub, geom='boxplot', col=reporter) +scale_y_log10(breaks=c(30,100,300,1000,3e3,10e3,30e3),labels=c(30,100,300,1000,3e3,10e3,30e3), limits=c(500,NA))+ylab('Intensity /au') + annotation_logticks(sides='l') + scale_colour_manual(values = c('myr-SNAPf'='blue', 'myr-4xSNAPf'='red')) + theme(text = element_text(size=20))+ theme(legend.justification=c(1,0), legend.position=c(1,0), legend.title=element_blank())
lp+ theme(legend.justification=c(1,0), legend.position=c(1,0), legend.title=element_blank())
```

```
# Seba experimenting with CLIP plots
mCLIPlh=subset(m[grep("CLIP",m$reporter),], region == "LH")
mCLIPlh$reporter = factor(mCLIPlh$reporter,levels=c("CD4-CLIPf","myr-4xCLIPf","myr-SNAPf","myr-4xSNAPf"))
kable(mCLIPlh)
```

|  | brain | name | id | foreground | background | fg.bsub | region | low | driver | reporter | insertion |
| --- | --- | --- | --- | --- | --- | --- | --- | --- | --- | --- | --- |
| 2 | BADF02 | RLH | 4 | 843.2256 | 355.6327 | 487.5928 | LH | /Volumes/JData/JPeople/Ben/Confocal/chemtag/BADF/nrrds/BADF02-Mz19-GJ851-UAS-CD4-CLIPf\_02.nrrd | MZ19 | CD4-CLIPf | GJ851 |
| 5 | BADF03 | RLH | 4 | 903.7537 | 479.4400 | 424.3137 | LH | /Volumes/JData/JPeople/Ben/Confocal/chemtag/BADF/nrrds/BADF03-Mz19-GJ851-UAS-CD4-CLIPf\_02.nrrd | MZ19 | CD4-CLIPf | GJ851 |
| 8 | BADF05 | RLH | 4 | 831.9586 | 435.4762 | 396.4824 | LH | /Volumes/JData/JPeople/Ben/Confocal/chemtag/BADF/nrrds/BADF05-Mz19-GJ851-UAS-CD4-CLIPf\_02.nrrd | MZ19 | CD4-CLIPf | GJ851 |
| 11 | BADF15 | RLH | 4 | 647.7242 | 345.9050 | 301.8191 | LH | /Volumes/JData/JPeople/Ben/Confocal/chemtag/BADF/nrrds/BADF15-Mz19-GJ851-UAS-CD4-CLIPf\_02.nrrd | MZ19 | CD4-CLIPf | GJ851 |
| 14 | BADH01 | RLH | 4 | 17703.0015 | 749.4748 | 16953.5267 | LH | /Volumes/JData/JPeople/Ben/Confocal/chemtag/BADH/nrrds/BADH01-mz19-15137-1-2M-UAS-myr-4xCLIPf-VK5\_02.nrrd | MZ19 | myr-4xCLIPf | VK5 |
| 17 | BADH11 | RLH | 4 | 11693.1782 | 489.6448 | 11203.5333 | LH | /Volumes/JData/JPeople/Ben/Confocal/chemtag/BADH/nrrds/BADH11-mz19-15137-1-2M-UAS-myr-4xCLIPf-VK5\_02.nrrd | MZ19 | myr-4xCLIPf | VK5 |
| 20 | BADH13 | RLH | 4 | 12008.2871 | 463.8511 | 11544.4360 | LH | /Volumes/JData/JPeople/Ben/Confocal/chemtag/BADH/nrrds/BADH13-mz19-15137-1-2M-UAS-myr-4xCLIPf-VK5\_02.nrrd | MZ19 | myr-4xCLIPf | VK5 |
| 23 | BADH15 | RLH | 4 | 11130.8039 | 489.6112 | 10641.1928 | LH | /Volumes/JData/JPeople/Ben/Confocal/chemtag/BADH/nrrds/BADH15-mz19-15137-1-2M-UAS-myr-4xCLIPf-VK5\_02.nrrd | MZ19 | myr-4xCLIPf | VK5 |
| 26 | BADS04 | RLH | 4 | 34953.6490 | 2187.7481 | 32765.9009 | LH | /Volumes/JData5/JPeople/Ben/Confocal/chemtag/BADS/nrrds/BADS04\_MZ19-15138-1-1M\_UAS-myr-4xCLIPf-attP40-CLIP-547\_02.nrrd | MZ19 | myr-4xCLIPf | P40 |
| 29 | BADS05 | RLH | 4 | 27734.9618 | 2639.6019 | 25095.3599 | LH | /Volumes/JData5/JPeople/Ben/Confocal/chemtag/BADS/nrrds/BADS05\_MZ19-15138-1-1M\_UAS-myr-4xCLIPf-attP40-CLIP-547\_02.nrrd | MZ19 | myr-4xCLIPf | P40 |
| 32 | BADS06 | RLH | 4 | 40600.6447 | 3003.4363 | 37597.2084 | LH | /Volumes/JData5/JPeople/Ben/Confocal/chemtag/BADS/nrrds/BADS06\_MZ19-15138-1-1M\_UAS-myr-4xCLIPf-attP40-CLIP-547\_02.nrrd | MZ19 | myr-4xCLIPf | P40 |
| 35 | BADS07 | RLH | 4 | 42306.2329 | 1706.5370 | 40599.6959 | LH | /Volumes/JData5/JPeople/Ben/Confocal/chemtag/BADS/nrrds/BADS07\_MZ19-15138-1-1M\_UAS-myr-4xCLIPf-attP40-CLIP-547\_02.nrrd | MZ19 | myr-4xCLIPf | P40 |
| 38 | BADS10 | RLH | 4 | 1433.8164 | 650.3232 | 783.4932 | LH | /Volumes/JData5/JPeople/Ben/Confocal/chemtag/BADS/nrrds/BADS10\_MZ19-GJ853-UAS-CD4-CLIPf-CLIP-547\_02.nrrd | MZ19 | CD4-CLIPf | GJ853 |
| 41 | BADS11 | RLH | 4 | 1851.5378 | 851.2291 | 1000.3087 | LH | /Volumes/JData5/JPeople/Ben/Confocal/chemtag/BADS/nrrds/BADS11\_MZ19-GJ853-UAS-CD4-CLIPf-CLIP-547\_02.nrrd | MZ19 | CD4-CLIPf | GJ853 |
| 44 | BADS13 | RLH | 4 | 689.4990 | 476.7031 | 212.7959 | LH | /Volumes/JData5/JPeople/Ben/Confocal/chemtag/BADS/nrrds/BADS13\_MZ19-GJ853-UAS-CD4-CLIPf-CLIP-547\_02.nrrd | MZ19 | CD4-CLIPf | GJ853 |

```
lp <- qplot(data=mCLIPlh, insertion, fg.bsub, geom='boxplot', col=reporter) +scale_y_log10(breaks=c(30,100,300,1000,3e3,10e3,30e3),labels=c(30,100,300,1000,3e3,10e3,30e3))+ylab('Intensity /au') + annotation_logticks(sides='l') + scale_colour_manual(values = c('CD4-CLIPf'='blue', 'myr-4xCLIPf'='red'))+ theme(text = element_text(size=20))+ theme(legend.justification=c(1,0), legend.position=c(1,0), legend.title=element_blank())
lp+ theme(legend.justification=c(1,0), legend.position=c(1,0), legend.title=element_blank())
```

```
# Seba experimenting with Halo plots

Halolh=subset(m[grep("Halo",m$reporter),], region == "LH")
Halolh$reporter = factor(Halolh$reporter,levels=c("CD4-CLIPf","myr-4xCLIPf","myr-Halo2","myr-SNAPf","myr-4xSNAPf","UAS-Halo7-CAAX","UAS-td3-Halo7-CAAX","UAS-td7-Halo7-CAAX"))
kable(Halolh)
```

|  | brain | name | id | foreground | background | fg.bsub | region | low | driver | reporter | insertion |
| --- | --- | --- | --- | --- | --- | --- | --- | --- | --- | --- | --- |
| 101 | BAFF01 | RLH | 4 | 390.9094 | 68.39040 | 322.5190 | LH | /Volumes/JData5/JPeople/Ben/Confocal/chemtag/BAFF/nrrds/BAFF01\_MZ19-UAS-Halo7-CAAX-P40-1\_02.nrrd | MZ19 | UAS-Halo7-CAAX | P40 |
| 104 | BAFF02 | RLH | 4 | 372.1502 | 65.75094 | 306.3992 | LH | /Volumes/JData5/JPeople/Ben/Confocal/chemtag/BAFF/nrrds/BAFF02\_MZ19-UAS-Halo7-CAAX-P40-1\_02.nrrd | MZ19 | UAS-Halo7-CAAX | P40 |
| 107 | BAFF03 | RLH | 4 | 418.7782 | 83.59495 | 335.1833 | LH | /Volumes/JData5/JPeople/Ben/Confocal/chemtag/BAFF/nrrds/BAFF03\_MZ19-UAS-Halo7-CAAX-P40-1\_02.nrrd | MZ19 | UAS-Halo7-CAAX | P40 |
| 110 | BAFF04 | RLH | 4 | 338.5232 | 75.40786 | 263.1153 | LH | /Volumes/JData5/JPeople/Ben/Confocal/chemtag/BAFF/nrrds/BAFF04\_MZ19-UAS-Halo7-CAAX-P40-1\_02.nrrd | MZ19 | UAS-Halo7-CAAX | P40 |
| 113 | BAFF05 | RLH | 4 | 765.7880 | 68.71237 | 697.0756 | LH | /Volumes/JData5/JPeople/Ben/Confocal/chemtag/BAFF/nrrds/BAFF05\_MZ19-UAS-Halo7-CAAX-P40-1\_02.nrrd | MZ19 | UAS-Halo7-CAAX | P40 |
| 116 | BAFF06 | RLH | 4 | 378.8666 | 66.89089 | 311.9757 | LH | /Volumes/JData5/JPeople/Ben/Confocal/chemtag/BAFF/nrrds/BAFF06\_MZ19-UAS-Halo7-CAAX-P40-1\_02.nrrd | MZ19 | UAS-Halo7-CAAX | P40 |
| 119 | BAFF07 | RLH | 4 | 314.6876 | 83.11179 | 231.5758 | LH | /Volumes/JData5/JPeople/Ben/Confocal/chemtag/BAFF/nrrds/BAFF07\_MZ19-UAS-Halo7-CAAX-P40-1\_02.nrrd | MZ19 | UAS-Halo7-CAAX | P40 |
| 122 | BAFF08 | RLH | 4 | 516.5100 | 102.41340 | 414.0966 | LH | /Volumes/JData5/JPeople/Ben/Confocal/chemtag/BAFF/nrrds/BAFF08\_MZ19-UAS-td3-Halo7-CAAX-P40-2\_02.nrrd | MZ19 | UAS-td3-Halo7-CAAX | P40 |
| 125 | BAFF09 | RLH | 4 | 528.0184 | 86.07839 | 441.9400 | LH | /Volumes/JData5/JPeople/Ben/Confocal/chemtag/BAFF/nrrds/BAFF09\_MZ19-UAS-td3-Halo7-CAAX-P40-2\_02.nrrd | MZ19 | UAS-td3-Halo7-CAAX | P40 |
| 128 | BAFF10 | RLH | 4 | 551.7946 | 111.35376 | 440.4409 | LH | /Volumes/JData5/JPeople/Ben/Confocal/chemtag/BAFF/nrrds/BAFF10\_MZ19-UAS-td3-Halo7-CAAX-P40-2\_02.nrrd | MZ19 | UAS-td3-Halo7-CAAX | P40 |
| 131 | BAFF11 | RLH | 4 | 543.0926 | 87.04913 | 456.0435 | LH | /Volumes/JData5/JPeople/Ben/Confocal/chemtag/BAFF/nrrds/BAFF11\_MZ19-UAS-td3-Halo7-CAAX-P40-2\_02.nrrd | MZ19 | UAS-td3-Halo7-CAAX | P40 |
| 134 | BAFF12 | RLH | 4 | 342.0960 | 65.19292 | 276.9030 | LH | /Volumes/JData5/JPeople/Ben/Confocal/chemtag/BAFF/nrrds/BAFF12\_MZ19-UAS-td3-Halo7-CAAX-P40-2\_02.nrrd | MZ19 | UAS-td3-Halo7-CAAX | P40 |
| 137 | BAFF13 | RLH | 4 | 468.0527 | 87.39650 | 380.6562 | LH | /Volumes/JData5/JPeople/Ben/Confocal/chemtag/BAFF/nrrds/BAFF13\_MZ19-UAS-td3-Halo7-CAAX-P40-2\_02.nrrd | MZ19 | UAS-td3-Halo7-CAAX | P40 |
| 140 | BAFF14 | RLH | 4 | 519.5695 | 102.73657 | 416.8330 | LH | /Volumes/JData5/JPeople/Ben/Confocal/chemtag/BAFF/nrrds/BAFF14\_MZ19-UAS-td3-Halo7-CAAX-P40-2\_02.nrrd | MZ19 | UAS-td3-Halo7-CAAX | P40 |
| 143 | BAFF15 | RLH | 4 | 563.3779 | 105.41412 | 457.9638 | LH | /Volumes/JData5/JPeople/Ben/Confocal/chemtag/BAFF/nrrds/BAFF15\_MZ19-UAS-td3-Halo7-CAAX-P40-2\_02.nrrd | MZ19 | UAS-td3-Halo7-CAAX | P40 |
| 146 | BAFF16 | RLH | 4 | 201.3274 | 44.01812 | 157.3093 | LH | /Volumes/JData5/JPeople/Ben/Confocal/chemtag/BAFF/nrrds/BAFF16\_MZ19-UAS-td7-Halo7-CAAX-P40-1\_02.nrrd | MZ19 | UAS-td7-Halo7-CAAX | P40 |
| 149 | BAFF17 | RLH | 4 | 286.3674 | 64.19660 | 222.1708 | LH | /Volumes/JData5/JPeople/Ben/Confocal/chemtag/BAFF/nrrds/BAFF17\_MZ19-UAS-td7-Halo7-CAAX-P40-1\_02.nrrd | MZ19 | UAS-td7-Halo7-CAAX | P40 |
| 152 | BAFF18 | RLH | 4 | 271.9601 | 61.75146 | 210.2087 | LH | /Volumes/JData5/JPeople/Ben/Confocal/chemtag/BAFF/nrrds/BAFF18\_MZ19-UAS-td7-Halo7-CAAX-P40-1\_02.nrrd | MZ19 | UAS-td7-Halo7-CAAX | P40 |
| 155 | BAFF19 | RLH | 4 | 227.8673 | 46.11702 | 181.7503 | LH | /Volumes/JData5/JPeople/Ben/Confocal/chemtag/BAFF/nrrds/BAFF19\_MZ19-UAS-td7-Halo7-CAAX-P40-1\_02.nrrd | MZ19 | UAS-td7-Halo7-CAAX | P40 |
| 158 | BAFF20 | RLH | 4 | 142.5434 | 28.58156 | 113.9618 | LH | /Volumes/JData5/JPeople/Ben/Confocal/chemtag/BAFF/nrrds/BAFF20\_MZ19-UAS-td7-Halo7-CAAX-P40-1\_02.nrrd | MZ19 | UAS-td7-Halo7-CAAX | P40 |
| 161 | BAFF21 | RLH | 4 | 192.9050 | 49.71022 | 143.1947 | LH | /Volumes/JData5/JPeople/Ben/Confocal/chemtag/BAFF/nrrds/BAFF21\_MZ19-UAS-td7-Halo7-CAAX-P40-1\_02.nrrd | MZ19 | UAS-td7-Halo7-CAAX | P40 |
| 164 | BAFF22 | RLH | 4 | 178.6128 | 59.01869 | 119.5942 | LH | /Volumes/JData5/JPeople/Ben/Confocal/chemtag/BAFF/nrrds/BAFF22\_MZ19-UAS-td7-Halo7-CAAX-P40-1\_02.nrrd | MZ19 | UAS-td7-Halo7-CAAX | P40 |
| 167 | BAFF23 | RLH | 4 | 236.7702 | 50.95907 | 185.8111 | LH | /Volumes/JData5/JPeople/Ben/Confocal/chemtag/BAFF/nrrds/BAFF23\_MZ19-UAS-td7-Halo7-CAAX-P40-1\_02.nrrd | MZ19 | UAS-td7-Halo7-CAAX | P40 |
| 170 | BAFF24 | RLH | 4 | 310.6939 | 64.75611 | 245.9377 | LH | /Volumes/JData5/JPeople/Ben/Confocal/chemtag/BAFF/nrrds/BAFF24\_MZ19-UAS-myr-Halo2-P40\_02.nrrd | MZ19 | myr-Halo2 | P40 |
| 173 | BAFF25 | RLH | 4 | 250.0917 | 46.05495 | 204.0368 | LH | /Volumes/JData5/JPeople/Ben/Confocal/chemtag/BAFF/nrrds/BAFF25\_MZ19-UAS-myr-Halo2-P40\_02.nrrd | MZ19 | myr-Halo2 | P40 |
| 176 | BAFF26 | RLH | 4 | 257.4146 | 50.51325 | 206.9014 | LH | /Volumes/JData5/JPeople/Ben/Confocal/chemtag/BAFF/nrrds/BAFF26\_MZ19-UAS-myr-Halo2-P40\_02.nrrd | MZ19 | myr-Halo2 | P40 |
| 179 | BAFF27 | RLH | 4 | 298.6770 | 51.77979 | 246.8972 | LH | /Volumes/JData5/JPeople/Ben/Confocal/chemtag/BAFF/nrrds/BAFF27\_MZ19-UAS-myr-Halo2-P40\_02.nrrd | MZ19 | myr-Halo2 | P40 |
| 182 | BAFF28 | RLH | 4 | 271.8469 | 48.49441 | 223.3525 | LH | /Volumes/JData5/JPeople/Ben/Confocal/chemtag/BAFF/nrrds/BAFF28\_MZ19-UAS-myr-Halo2-P40\_02.nrrd | MZ19 | myr-Halo2 | P40 |
| 185 | BAFF29 | RLH | 4 | 314.4700 | 55.96135 | 258.5086 | LH | /Volumes/JData5/JPeople/Ben/Confocal/chemtag/BAFF/nrrds/BAFF29\_MZ19-UAS-myr-Halo2-P40\_02.nrrd | MZ19 | myr-Halo2 | P40 |
| 188 | BAFF30 | RLH | 4 | 282.4809 | 51.66062 | 230.8202 | LH | /Volumes/JData5/JPeople/Ben/Confocal/chemtag/BAFF/nrrds/BAFF30\_MZ19-UAS-myr-Halo2-P40\_02.nrrd | MZ19 | myr-Halo2 | P40 |

```
lp <- qplot(data=Halolh, insertion, fg.bsub, geom='boxplot', col=reporter) +scale_y_log10(breaks=c(30,100,300,1000,3e3,10e3,30e3),labels=c(30,100,300,1000,3e3,10e3,30e3))+ylab('Intensity /au') + annotation_logticks(sides='l') + scale_colour_manual(values = c("UAS-Halo7-CAAX"='blue',"UAS-td3-Halo7-CAAX"='red',"UAS-td7-Halo7-CAAX"='green',"myr-Halo2"='orange'))+ theme(text = element_text(size=20))+ theme(legend.justification=c(1,0), legend.position=c(1,0), legend.title=element_blank())
lp+ theme(legend.justification=c(1,0), legend.position=c(0.5,0), legend.title=element_blank())
```

Show table of LH results

```
LH_results <- gm[gm$region=="LH",c('reporter', 'insertion','val')]
dfLH <- data.frame(rbind(LH_results))
kable(LH_results)
```

| reporter | insertion | val |
| --- | --- | --- |
| CD4-CLIPf | GJ851 | 403 |
| CD4-CLIPf | GJ853 | 666 |
| myr-4xCLIPf | P40 | 34015 |
| myr-4xCLIPf | VK5 | 12586 |
| myr-4xSNAPf | P40 | 8627 |
| myr-4xSNAPf | VK5 | 12626 |
| myr-Halo2 | P40 | 231 |
| myr-SNAPf | P2 | 916 |
| myr-SNAPf | P40 | 1700 |
| UAS-Halo7-CAAX | P40 | 353 |
| UAS-td3-Halo7-CAAX | P40 | 411 |
| UAS-td7-Halo7-CAAX | P40 | 167 |

Use LH\_results to generate ratios using the mean of the old version of each reporter to compare with each insertion fo the new reporters

```
CLIPf <- mean(dfLH[1:2,3])
x4CLIPfp40 <- dfLH[3,3]
x4CLIPfvk5 <- dfLH[4,3]
SNAPf <- mean(dfLH[8:9,3])
x4SNAPfp40 <- dfLH[5,3]
x4SNAPfvk5 <- dfLH[6,3]
Halo2 <- dfLH[7,3]
Halo7 <- dfLH[10,3]
x3Halo7 <- dfLH[11,3]
x7Halo7 <- dfLH[12,3]
comparisons <- c("v1CLIPf vs 4xCLIPfp40",
    "v1CLIPf vs 4xCLIPfvk5",
    "v1SNAPf vs 4xSNAPfp40",
    "v1SNAPf vs 4xSNAPfvk5",
    "Halo2 vs Halo7",
    "Halo2 vs 3xHalo7",
    "Halo2 vs 7xHalo7")
ratios <- c((x4CLIPfp40/CLIPf),(x4CLIPfvk5/CLIPf),
    (x4SNAPfp40/SNAPf),(x4SNAPfvk5/SNAPf),
    (Halo7/Halo2),(x3Halo7/Halo2),(x7Halo7/Halo2))
percentchange <- c((((x4CLIPfp40-CLIPf)/CLIPf)*100),
    (((x4CLIPfvk5-CLIPf)/CLIPf)*100),
    (((x4SNAPfp40-SNAPf)/SNAPf)*100),
    (((x4SNAPfvk5-SNAPf)/SNAPf)*100),
    (((Halo7-Halo2)/Halo2)*100),
    (((x3Halo7-Halo2)/Halo2)*100),
    (((x7Halo7-Halo2)/Halo2)*100))
LH_ratios <- data.frame(comparisons,ratios,percentchange)
colnames(LH_ratios) <- c("Comparisons","Ratio","Percentage Change")
kable(LH_ratios)
```

| Comparisons | Ratio | Percentage Change |
| --- | --- | --- |
| v1CLIPf vs 4xCLIPfp40 | 63.6389149 | 6263.89149 |
| v1CLIPf vs 4xCLIPfvk5 | 23.5472404 | 2254.72404 |
| v1SNAPf vs 4xSNAPfp40 | 6.5955657 | 559.55657 |
| v1SNAPf vs 4xSNAPfvk5 | 9.6529052 | 865.29052 |
| Halo2 vs Halo7 | 1.5281385 | 52.81385 |
| Halo2 vs 3xHalo7 | 1.7792208 | 77.92208 |
| Halo2 vs 7xHalo7 | 0.7229437 | -27.70563 |
| END |  |  |
